# Daisy-chain gene drives: The role of low cut-rate, resistance mutations, and maternal deposition

**DOI:** 10.1371/journal.pgen.1010370

**Published:** 2022-09-19

**Authors:** Sebald A. N. Verkuijl, Michelle A. E. Anderson, Luke Alphey, Michael B. Bonsall

**Affiliations:** 1 Department of Biology, University of Oxford, Oxford, United Kingdom; 2 Arthropod Genetics, The Pirbright Institute, Pirbright, United Kingdom; Peking University, CHINA

## Abstract

The introgression of genetic traits through gene drive may serve as a powerful and widely applicable method of biological control. However, for many applications, a self-perpetuating gene drive that can spread beyond the specific target population may be undesirable and preclude use. Daisy-chain gene drives have been proposed as a means of tuning the invasiveness of a gene drive, allowing it to spread efficiently into the target population, but be self-limiting beyond that. Daisy-chain gene drives are made up of multiple independent drive elements, where each element, except one, biases the inheritance of another, forming a chain. Under ideal inheritance biasing conditions, the released drive elements remain linked in the same configuration, generating copies of most of their elements except for the last remaining link in the chain. Through mathematical modelling of populations connected by migration, we have evaluated the effect of resistance alleles, different fitness costs, reduction in the cut-rate, and maternal deposition on two alternative daisy-chain gene drive designs. We find that the self-limiting nature of daisy-chain gene drives makes their spread highly dependent on the efficiency and fidelity of the inheritance biasing mechanism. In particular, reductions in the cut-rate and the formation of non-lethal resistance alleles can cause drive elements to lose their linked configuration. This severely reduces the invasiveness of the drives and allows for phantom cutting, where an upstream drive element cuts a downstream target locus despite the corresponding drive element being absent, creating and biasing the inheritance of additional resistance alleles. This phantom cutting can be mitigated by an alternative indirect daisy-chain design. We further find that while dominant fitness costs and maternal deposition reduce daisy-chain invasiveness, if overcome with an increased release frequency, they can reduce the spread of the drive into a neighbouring population.

## Author summary

Reducing the harm of pest species by the introgression of traits into a wild population is often limited by the difficulties of mass rearing and release of modified individuals. Gene drives present an opportunity to substantially reduce the release frequencies required to spread a particular modification. However, uniform modification of a target species is, with a few specific exceptions, not necessary or desirable. Self-limiting gene drives, such as daisy-chain gene drives, have been widely discussed as a potential solution, allowing the invasiveness of a drive release to be tuned to the target population. Here, we investigate through computational modelling how daisy-chain gene drives perform when subjected to commonly observed inefficiencies associated with CRISPR-Cas9-based inheritance biasing. Compared to a self-perpetuating drive, daisy-chain gene drives are sensitive to factors that cause their separate elements to segregate prematurely. In particular, a reduction in the DNA cut-rate and an increase in the formation of resistance alleles. We find that the effect of inefficiencies in the drive mechanism is generally more pronounced when the drive is at low frequencies. With low rates of migration, this substantially reduces daisy-chain gene drives spread into a neighbouring non-target population.

## Introduction

Synthetic gene drive methods are potentially powerful approaches in the management of agricultural pests and disease vectors. However, for many applications, the uniform modification of a species by a fully self-perpetuating drive is unnecessary and likely undesirable [1]. From an ecological, regulatory, and public acceptance perspective, a drive that is spatially and temporally limited may be more appropriate. Gene drives can be inherently self-limiting, but this is generally due to low inheritance biasing efficiency, high fitness costs, or frequency-dependent effects, all of which can reduce their practical use. Some self-limiting systems have been proposed that are restricted in specific ways that maintain efficient but local transgene invasion [2]. These include targeting specific DNA sequences found only in subpopulations [3], dependence on supplementation with a synthetic inducer molecule [4, 5], and split drive systems.

In a split drive system, at least one genetic component essential for the gene drive inheritance biasing mechanism is removed from the biased locus and is located elsewhere in the genome [6]. The split drive will bias, or drive, a primary locus; however, those copies cannot bias themselves in subsequent generations without the component(s) from the unbiased (non-driving) secondary locus. As the split drive spreads into a large population, the lack of amplification of the secondary locus will limit the rate of further amplification of the primary drive element. Compared to a cargo-only release (inundative release), split-drives provide a modest reduction in the number of modified individuals that need to be released to spread a trait among a wild population. Researchers have sought to develop variations on the basic split drive design with increased invasiveness, but that can still be tuned to specific target populations and applications.

For a meiotic drive system that biases chromosomal regions proportional to their recombination distance, the split drive elements can be located an intermediate recombination distance from each other. This intermediate linkage causes most, but crucially not all, progeny that inherit the main element to also inherit the secondary element, increasing invasiveness, tunable by the recombination distance [7]. However, the most extensively studied gene drives are homing endonuclease gene drives (HEGs) that, with poorly understood exceptions [8], only bias a very limited genomic region. Therefore, it is unlikely that the intermediate inheritance biasing of a separate split drive element can be incorporated in the same way. For HEGs, other split drive strategies have been proposed that would allow the spread to be tuned (and are also applicable to a meiotic drive system); one of these is the daisy-chain gene drive [9].

Daisy-chain gene drives (DCDs) sequentially link drive elements to prolong inheritance bias on a main effector modification, which is the final element in the chain. Each element biases the inheritance of another element apart from the final element, and each element is biased except for the first element. Sequential reduced copies (reduced by one element) of the released drive configuration will continue to bias an effector modification until copies are produced where it is the only element remaining. Increasing the number of elements in the chain prolongs the generations of inheritance bias experienced by the main effector modification. DCDs are a seemingly safer alternative to most applications for which a self-perpetuating drive would be considered. However, the increased complexity of their design raises questions about the efficacy of the molecular mechanism required for practical implementation.

Engineered HEGs with varying inheritance biasing efficiencies have been reported in various species [6, 10–16], most commonly with the CRISPR-Cas9 nuclease. CRISPR-Cas9 is a two-component endonuclease formed by the Cas9 protein and a guide RNA (gRNA) that specifies the sequence to be cut [17]. CRISPR-Cas9-based homing split drives can be engineered by moving either the gRNA gene(s) or the Cas9 nuclease gene, as both are essential for a cut to occur. DNA breaks that Cas9 makes can, under specific circumstances, induce cellular repair pathways to copy a gene drive from one homologous chromosome to another (homing). This results in the biased genetic element converting itself from a heterozygous state to a homozygous state.

There are several ways in which the HEG inheritance biasing mechanism may not work as intended. The most straightforward is a less than saturating cut-rate of the target allele. A common optimisation strategy for HEGs is to test endogenous regulatory sequences that limit nuclease expression to meiotic cells, which are expected to perform homing more readily after a DNA break. There may be cases where regulatory elements with the best expression window may not have the optimal expression level. An example of this is the report that in mice, the cotranslation of Cas9 with an endogenous gene resulted in a more favourable DNA repair profile compared to previous efforts with an autonomously expressed Cas9 [15, 18]; however, this improved DNA repair profile came at the cost of the overall cut-rate [19]. When fitness costs are low, self-perpetuating drives are generally robust to lower cut-rates as they can simply try again in a subsequent generation [20]. However, for DCDs, their self-limiting mechanism continues to operate, and the unbiased drive elements can be expected to randomly segregate, leading to only a subset of progeny co-inheriting all the drive elements. As such, even if the overall allele frequency in the population remains unchanged, there will be less co-occurrence of the multiple drive elements necessary for inheritance bias. Moreover, for DCDs, individuals may emerge with one or more upstream elements without the corresponding downstream drive element. Certain DCD designs will continue to cut and bias their downstream locus even when the associated drive element is not present to serve as the repair template on the homologous chromosome (phantom cutting).

The inheritance biasing of HEGs relies on homology-directed repair (HDR) to mediate copying of the drive element(s). However, cells have multiple competing pathways that can resolve a DNA break. Sequence changes made by alternative DNA repair pathways (and specific undesired HDR events) can be grouped by their consequences into two categories: type-1 and type-2 resistance mutations [21]. Type-1 resistance mutations (r1) are changes in DNA sequence that disrupt the gRNA binding site while preserving the normal function of the target gene (silent mutations). Type-2 resistance mutations (r2) also prevent the nuclease from cutting the site and, in addition, disrupt the normal function of the target gene (nonsense/missense mutations). Generally, target genes and specific target sites within those genes are chosen such that type-2 resistance alleles impose the same or higher fitness costs than the drive element. Type-2 resistance alleles will be selected against and will not prevent the drive element from approaching fixation. However, type-1 resistance mutations are expected to impose no or substantially lower fitness costs than the drive element. Even if type-1 resistance alleles are produced only very rarely, positive selection can increase their frequency and prevent the drive element from reaching the frequency needed for its intended function [22]. The segregation of drive elements with downstream resistance alleles and additional biasing of these resistance alleles presents a unique challenge to DCDs that has not been investigated.

Lastly, another common HEG attribute is deposition, which may also be expected to have unique interactions with DCDs. Deposition is the phenomenon in which drive components expressed or translated in a parent can persist into their offspring. This can occur even if the offspring does not inherit the gene from which those components were expressed in the parent. Deposition occurs almost exclusively from female drive carriers (maternal deposition), with the expression pattern of the nuclease found to influence the degree and pattern of deposition observed [12, 14, 16, 18, 21–33]. Deposition can result in nuclease activity at times when the inheritance biasing process is not favoured and in individuals who do not carry the drive, generating additional resistance mutations. Furthermore, nuclease activity early in development can cause the conversion of somatic tissues creating (mosaic) individuals with additional fitness costs. In contrast, if those genotypes with additional fitness costs are generated in the germline by autonomous expression of the drive, these costs generally only manifest in the next generation.

Although some modelling has been done of DCDs [9, 34–36], limited research has been done on understanding their interaction with functional resistance alleles, recessive and dominant fitness costs, reduced cut-rates, and maternal deposition. Here, we investigate, through theoretical modelling, the performance of two alternative homing endonuclease DCD designs when subjected to the common drive imperfections introduced above. In each case, we compare their performance to a self-perpetuating single-element drive allowing us to highlight unique interactions that affect DCDs. Furthermore, we evaluate the degree to which DCDs remain limited to the target population and do not spread substantially to a secondary population linked by migration. We find that DCDs place much higher requirements on the fidelity and efficiency of the drive mechanism for their spread. In addition, we find that phantom cutting can severely increase the production of type-1 resistance alleles, but this can be partially mitigated by using an indirect DCD design.

## Results

### Phantom cutting by DCDs can bias type-1 resistance alleles

**Fig 1:**
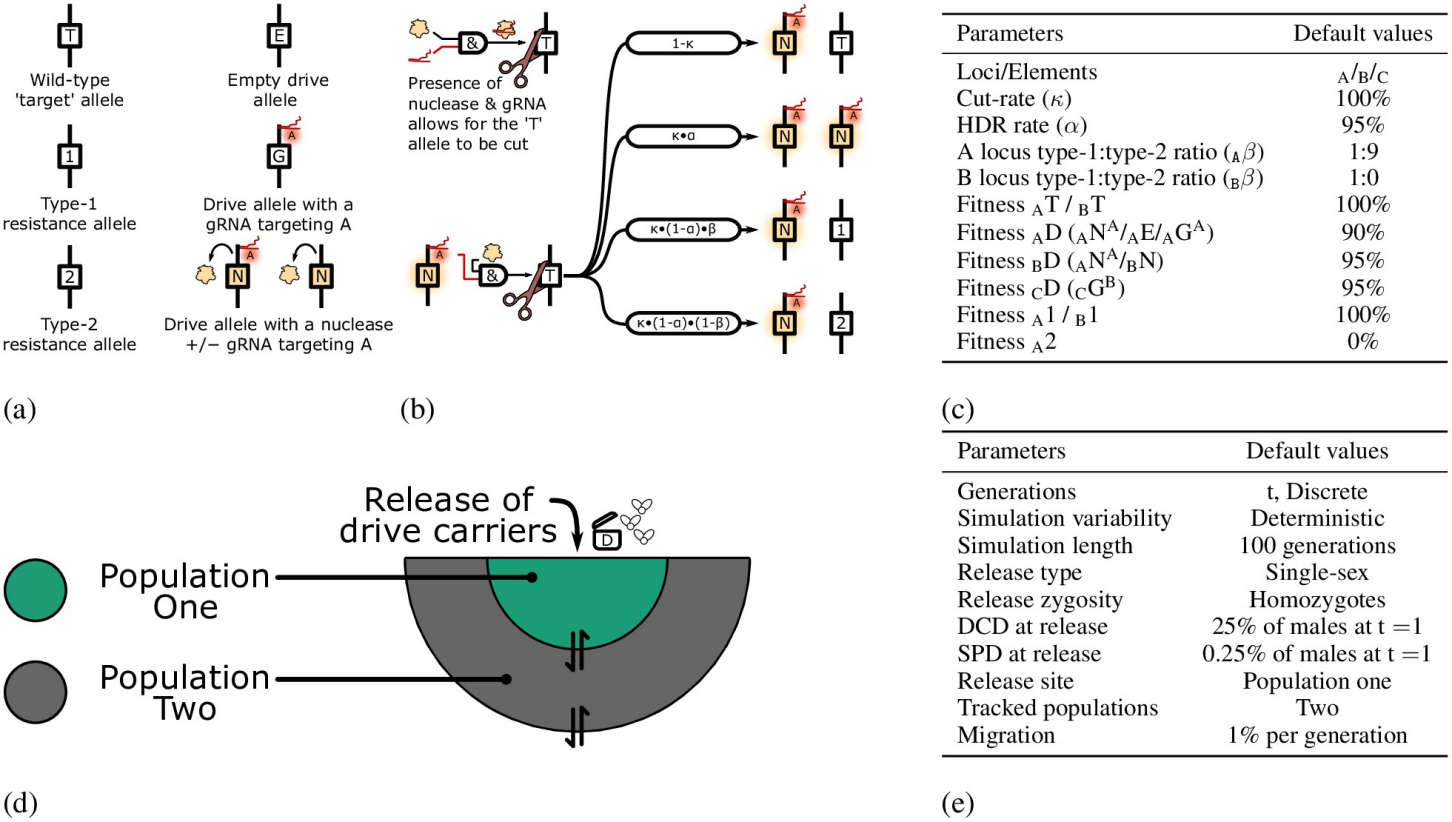
Drive nomenclature system and overview of simulation parameters. **(a)** Different alleles considered in our computational simulations: Wild-type target (T), type-1 resistance (1), type-2 resistance (2), and drive alleles (E, G, and N: collectively D). **(b)** When a target allele, a gRNA targeting that specific allele, and the nuclease are present in the same genotype, there is a certain likelihood that a cut occurs (by default 100%). The cut allele can be repaired by interhomolog HDR, or to a type-1 resistance allele or type-2 resistance allele. **(c)** Default nuclease and fitness parameters. The A drive allele has a fitness of 90% to account for general fitness costs (e.g., metabolic) and imperfect rescue of the haploinsufficient target gene by the drive element. This is the only fitness cost of the self-perpetuating gene drive. For DCDs, the B and C drive alleles have a fitness of 95%. Each fitness cost is dominant, with no additional recessive cost unless specifically listed for that simulation. Individuals carrying one or more of each different DCD allele are therefore assumed to have a fitness of 81% (90% ⋅ 95% ⋅ 95%). Type-2 resistance alleles at the A locus are assumed to be dominant lethal (fitness 0%). The B element is in a neutral locus; as such, its resistance alleles are all neutral and classified as type-1. The C locus is never cut, and resistance alleles are not generated at that locus. **(d)** Illustration of the two linked populations for which we report the allele frequency. Drive individuals are released into population one at the start of the simulation. Each generation, 1% of the surviving adults migrate to the neighbouring population. Population one receives 1% of population two, while population two receives 0.5% of population one and 0.5% of exclusively wild-types. **(e)** Default release frequency and simulation length parameters. DCD: Daisy-Chain Drive. SPD: Self-Perpetuating Drive.

We simulate homing endonuclease gene drives in two populations with up to three unlinked diploid loci designated by a subscript: _A_, _B_, or _C_. Briefly, a wild-type target allele (T) can be cut by the combined product of a nuclease allele (N) and a gRNA. The nuclease is target independent, whereas the gRNA always matches a specific wild-type target allele. The presence of a gRNA gene in an allele is indicated by a superscript of the locus to which it is targeted and can be together with a nuclease gene (N^A^) or only a gRNA gene without the nuclease gene (G^A^). A drive element can also lack a Cas9 or gRNA gene (E), being empty apart from the cargo gene/effector modification that is being spread. The nomenclature system we use is further expanded in Fig 1a.

Nuclease deposition into the embryo, differences in fitness, and nuclease expression in the germline can cause a change in the genotype frequency from one discrete generation to the next (Fig 1b and S1 Fig). The fitness and drive conversion rates for the many unique combinations of alleles are extrapolated from a small set of parameters listed in Fig 1c. The inheritance biasing parameters most closely resemble those of HEGs developed in the *Anopheles* mosquito [25, 30]. However, we assume that type-2 resistance alleles are dominant lethal at the A locus (haplolethal target gene) and the drive elements at that locus carry a rescue element [6, 25, 26]. The creation of a rescue element is generally achieved by including a recoded version (many synonymous codon substitutions) of the target site and downstream portions of the target gene. Essentially, engineering the drive allele to carry extensive type-1 resistance mutations. As such, we assume that a relatively high fraction of non-HDR repair results in type-1 resistance alleles (10% of non-HDR events, 0.5% overall) when compared to what has been achieved targeting a highly conserved locus (for which creating a viable rescue element may not be possible) [37].

For each simulation, we report the zygote genotype frequency before fitness, deposition, and expression-mediated changes in genotype frequency occur in that generation. We track genotype frequencies in two separate populations which have a 1% migration rate, with population two receiving a constant migration of wildtypes from the general population in addition to migration to and from population one (Fig 1d). Alleles for a particular population are indicated with an additional number in the subscript (e.g., _A1_). Unless otherwise specified, our default release scenario for daisy-chain drives is such that at the start of the simulation, 25% of males (12.5% overall allele frequency) in population one are homozygous for all drive elements (Fig 1e). The self-perpetuating drive is initiated with a frequency of male drive carriers 100 times lower (0.25%) compared to the release scenario for DCDs.

**Fig 2:**
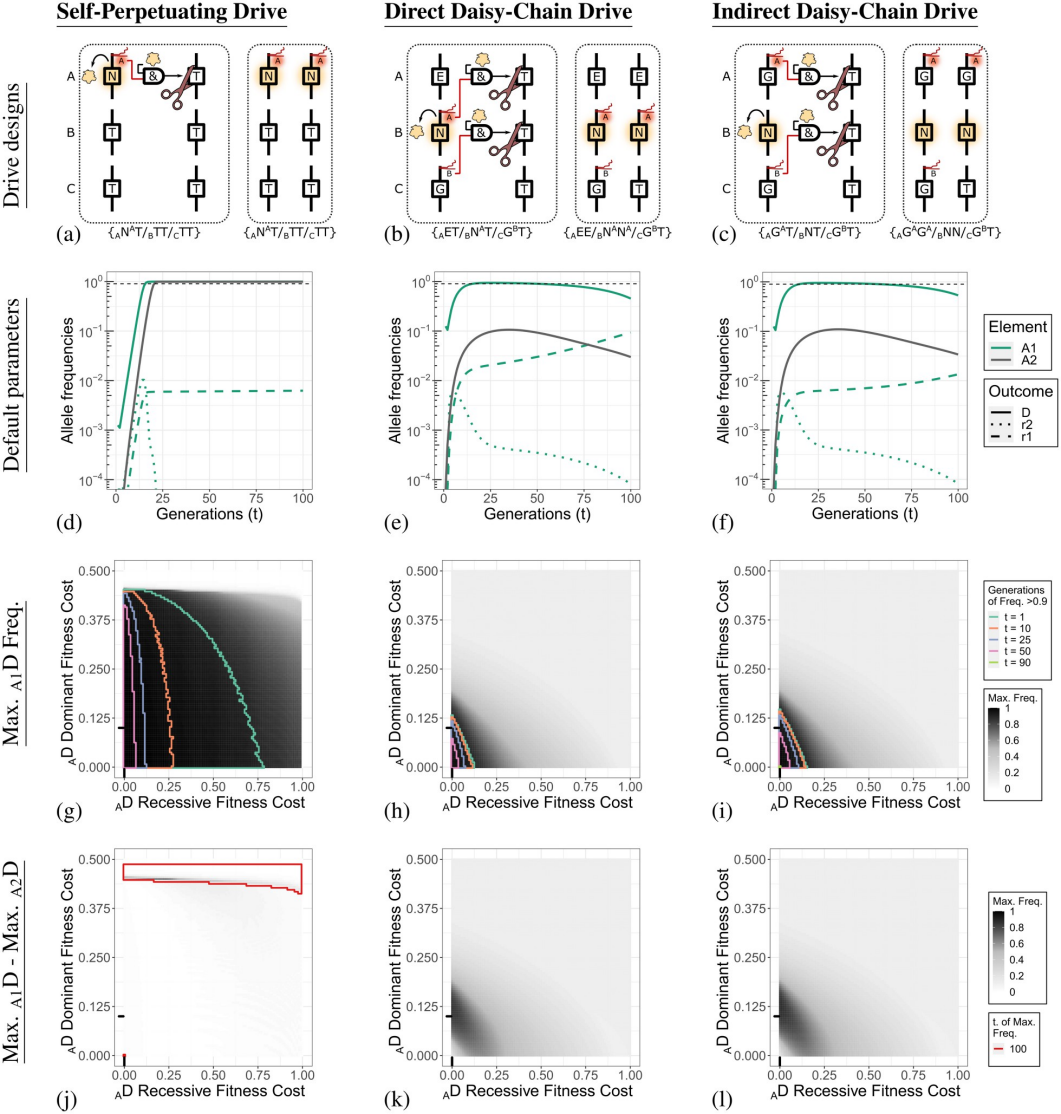
Illustration of drive designs, default behaviour, and performance when subjected to different recessive and dominant _A_D fitness costs. **Column 1**. Self-Perpetuating Drive. **Column 2**. Direct Daisy-Chain Drive. **Column 3**. Indirect Daisy-Chain Drive. **Row 1 (a-c)**. Illustration of the molecular designs of the three drives we simulate. Each design is shown as heterozygote, with on the right the product resulting from inheritance bias. **Row 2 (d-f)**. A locus allele dynamics under the default conditions. The thin dashed line indicates a frequency of 90%. An allele at locus A in population one is indicated by _A1_ (green lines), and a drive allele at locus A in population two is indicated by _A2_ (gray line). Type 1 and 2 resistance alleles are not shown for population two in these panels. The allele dynamics for all loci are shown in S2 Fig. **Row 3 (g-i)**. Parameter sweep for different recessive and dominant fitness costs applied to the _A_D (_A_N^A^/_A_E/_A_G^A^) allele. The shading of the heat map indicates the maximum _A_D allele frequency in population one reached within the 100 generations simulated. The maximum frequency of _A_1 alleles and outcomes for population two are shown in S7 Fig. An immediate decline after release results in the maximum frequency being the release frequency. Regions are boxed with coloured lines that indicate threshold values for the number of generations in which the _A1_D allele frequency was greater than 90%. The black lines on each axis indicate the default value for the parameter being varied. **Row 4 (j-l)**. Difference in the maximum _A_D allele frequency between population one and two. Regions boxed with a red line are simulations in which the maximum allele frequency of population one or two was at generation 100 and the outcomes may change if additional generations are simulated.

The three different HEG designs that we investigate are a single-element self-perpetuating drive (_A_N^A^) shown in Fig 2a and two different three-locus daisy-chain drives. For each drive, the A drive element is inserted into and rescues a haploinsufficient endogenous gene. For DCDs, the B and C elements are located at neutral sites. The first DCD design we call a direct DCD (_A_E/_B_N^A^/_C_G^B^), shown in Fig 2b and is the most similar to the design modelled by Noble et al. [9]. The C element (_C_G^B^) carries a gRNA targeting _B_T. The B element (_B_N^A^) carries both a nuclease and a gRNA targeting _A_T. The drive element for A would, in practice, contain the desired effector modification, but does not contain any components (gRNA or nuclease) that are directly involved in inheritance biasing (denoted as _A_E without a superscript).

The second DCD design we consider is an indirect DCD (_A_G^A^/_B_N/_C_G^B^), shown in Fig 2c, which has the gRNA targeting the A element expressed from the A element itself (_A_G^A^). The chain structure is maintained as the A element will only function in the presence of the nuclease expressed from the B element (_B_N). The C element is identical in both DCD designs. The indirect DCD has several practical benefits, including that A and B element transgenic lines can be developed independently, while for the direct DCD design, the A targeting gRNA(s) located on B limits interoperability. A DCD design functionally similar to this (using orthogonal nucleases) was proposed in Noble 2019 et al., but not explicitly investigated [9].

The allele dynamics of the self-perpetuating drive (_A_N^A^) shown in Fig 2d represents the maximally efficient implementation of an HEG, albeit with unlimited spread. The A drive element (the only drive element) in population one (_A1_D) reaches a maximum allele frequency of 99.4% at generation 23. The fitter type-1 alleles slowly outcompete the drive allele, but this process will take many generations, since at generation 23 the type-1 resistance allele (_A1_1) frequency is only 0.6%. The drive element in population two reaches a maximum frequency of 98.8% at generation 24. While the initial spread of the drive element into population two lags a few generations behind that of population one, migration means that population one only reaches its maximum drive frequency when population two is also (almost) at its maximum drive frequency. The frequency of wild-type alleles in population two quickly drops and stabilises at just over 0.55%. This is due to the 0.5% migration of wild-type individuals from the general population each generation and the reduced fitness of drive-carrying individuals (90% of wildtype).

While the allele frequency dynamics of the B and C alleles are almost identical for the two DCD designs (S2 Fig), the A allele dynamics, especially the type-1 resistance allele dynamics, differs substantially (Fig 2e and 2f). The direct DCD reaches a maximum _A1_E frequency of 93.8% at generation 25, which coincides with a type-1 resistance allele frequency of 2.1%. The maximum _A2_E frequency in population two is 10.6% at generation 35. The indirect DCD reaches a maximum _A1_G^A^ frequency of 95.3% at generation 27, which coincides with a type-1 resistance allele frequency of 0.6%. The maximum _A2_G^A^ frequency in population two is 11% at generation 36.

The difference in performance between the two DCD designs occurs due to a phenomenon we term phantom cutting, illustrated in S3 Fig. This occurs when an upstream element, such as the direct DCD B element(_B_N^A^), attempts to bias the inheritance of the downstream A element, even when the downstream element is not present. This is possible in our simulation as with a homing rate less than 100% the B drive element is occasionally co-inherited with a resistance allele generated at the A locus. Phantom cutting is only possible for the direct DCD as both the A gRNA and the nuclease are on B (_B_N^A^) and it does not occur with the indirect DCD, as the nuclease on the B element (_B_N) requires the gRNA expressed from the A element drive allele (_A_G^A^).

We have assumed an HDR rate of 95% and a type-1 to type-2 resistance allele repair ratio of 1:9 at the A locus, resulting in 0.5% of DNA breaks generating type-1 alleles. In the indirect design, these type-1 resistance alleles persist and slowly outcompete the drive alleles but are not otherwise biased in any way. In the direct DCD, these type-1 resistance alleles can be biased in the same way that the A drive allele can (e.g., _A_1T/_B_N^A^T→ _A_11/_B_N^A^T), and therefore reach much higher frequencies. In our simulations, type-2 resistance alleles at the A locus cannot be biased by phantom cutting as even one copy is dominant lethal. Phantom cutting can only occur when there are _A_T alleles remaining in the population. As the A drive and resistance alleles replace the _A_T alleles, the frequency of phantom cutting events fades away.

The default single-sex drive frequency at release of 25% allows the daisy-chains to spread to high frequencies in population one, but not in population two. As such, the release frequency can be considered to be roughly tuned to the drive’s inheritance biasing efficiency, fidelity, and fitness. The self-perpetuating drive cannot be tuned and will spread to high frequencies in both populations. The A locus drive and type-1 allele frequency dynamics are shown for different HDR and migration rates in S4 and S5 Figs, respectively.

It is important to note that the differences between the two DCD designs depend on the assumptions we have made compared to previous work. Using the parameters and assumptions of Noble 2019 et al. [9] (100% cut-rate, no type-1 alleles, and type-2 alleles at A are dominant lethal), the direct and indirect DCD designs behave identically to each other (S6 Fig, columns one and two). Phantom cutting cannot occur because no progeny survive in which the B element segregates away from the A element. Moreover, the DCD designs that we evaluate differ from other models of DCDs [9, 35] in that they only have a nuclease expressed by the B element, not by both the B and C element (i.e., _A_E/_B_N^A^/_C_N^B^). With a nuclease on the C element, the direct design simulations match those reported by Noble et al. [9] (S6 Fig, columns three and four). For the indirect DCD design, the nuclease expressed from the C element helps increase the spread of _A_G^A^, but also generates many more resistance alleles at the B locus due to phantom cutting. All other simulations in this study use DCD designs without a nuclease on the C element.

Fig 2g–2i show the maximum frequency of the A drive element, and the number of generations it remains above a 90% allele frequency under different recessive and dominant _A_D fitness costs. The recessive fitness costs apply to individuals with two copies of the drive, the dominant fitness costs apply equally to individuals with one or two copies. Obviously, minimisation of the fitness costs is the most conducive to DCD spread. However, without adjusting the release frequency, this also allows the drive allele to spread beyond population one and reach high frequencies in population two (S7 Fig). In Fig 2j–2l we contrast the two populations by subtracting the maximum drive frequency in population two from the maximum drive frequency in population one. This reveals a more narrow parameter space in which the DCDs can spread to high frequencies in the target population, yet do not substantially invade population two. We have highlighted cases where the maximum drive frequency in either population coincides with the end of the simulation, indicating that simulating more generations may change the outcomes.

With a fixed release frequency, dominant fitness costs appear to be more desirable when evaluating the differential spread in the two populations. Unlike recessive fitness costs, dominant fitness costs do not additionally penalise the drive for spreading to a high frequency in population one and immediately apply to the drive alleles that have migrated to population two, even when they are at low frequencies. Furthermore, dominant fitness costs can apply to heterozygous individuals carrying a type-1 resistance allele. Individuals with a type-1 allele are never affected by a drive with an exclusively recessive fitness cost, increasing the relative benefit for an individual carrying such a functional resistance allele.

The relatively low default fitness costs of the A drive rescue element make these simulations representative of low fitness cost population modification drives (e.g., spreading an antibody gene in a vector of human disease). Another class of drives is aimed at population suppression, with the goal of reducing a target species’s population density. We have performed simulations at different release frequencies for a recessive lethal (S8 Fig), and a sex-specific recessive lethal (S9 Fig) A element target gene. In both cases, the A element does not contain a rescue gene and imposes the same fitness cost as a type-2 resistance allele (i.e., disrupts the target gene as it homes). As may be expected, the self-perpetuating drive approaches fixation under the broadest set of conditions, largely independent of the release frequency. All drives rapidly decrease in frequency after reaching their maximum, with type-1 resistance alleles driven to near-fixation by the self-perpetuating drive (type-1 alleles would reach fixation were it not for the low levels of migration of wildtypes from the general population). The DCDs only approach a maximum drive frequencies similar to the self-perpetuating drive with upward of 70% of males carrying the DCD at the start of the simulation. In all cases, the indirect design is burdened by fewer type-1 mutations and spreads to higher frequencies.

Together, these results show that even low rates of type-1 resistance allele generation cause the two DCDs to perform differently from each other. Furthermore, the maximum frequency achieved by a DCD is highly dependent on its fitness profile, with dominant fitness costs providing the largest difference between the spread in population one and two.

### DCDs are severely impacted by reductions in cut-rate

**Fig 3:**
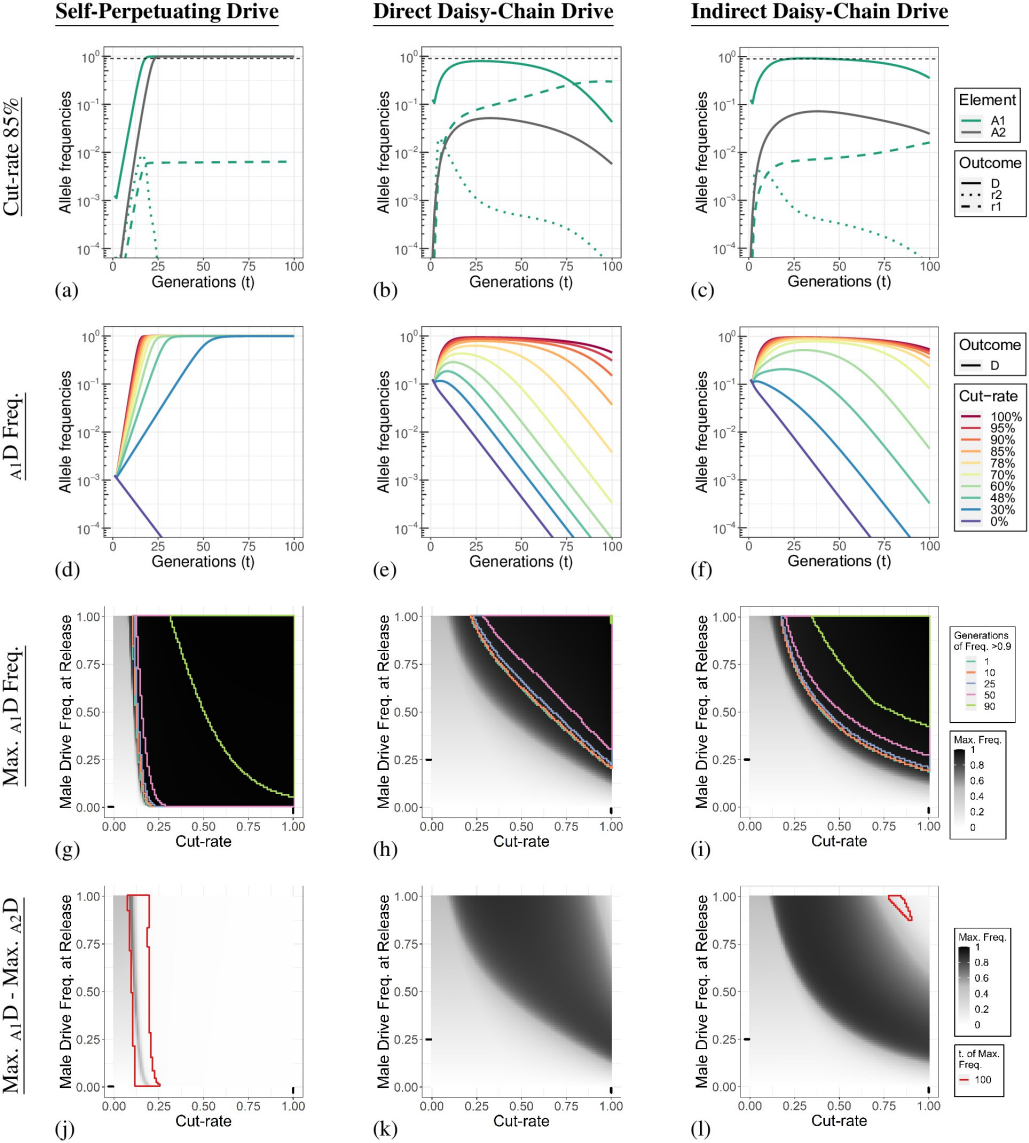
Daisy-chain gene drive element prematurely segregate with reduced cut-rates. **Column 1**. Self-Perpetuating Drive. **Column 2**. Direct Daisy-Chain Drive. **Column 3**. Indirect Daisy-Chain Drive. **Row 1 (a-c)**. Individual allele dynamics with a cut-rate of 85%. The thin dashed line indicates a frequency of 90%. An allele at locus A in population one is indicated by _A1_ (green lines), and a drive allele at locus A in population two is indicated by _A2_ (gray line). Type 1 and 2 resistance alleles are not shown for population two in these panels. The allele dynamics for all loci are shown in S10 Fig. **Row 2 (d-f)**. A element drive allele dynamics for population one with different cut-rates. Allele dynamics of the A locus drive allele in population two, and type-1 resistance alleles for both populations are shown in S11 Fig. **Row 3 (g-i)**. Parameter sweep for the male drive frequency at release and germline cut-rate. The shading of the heat map indicates the maximum _A_D allele frequency in population one reached within the 100 generations simulated. The maximum frequency of _A_1 alleles and outcomes for population two are shown in S13 Fig. An immediate decline after release results in the maximum frequency being the release frequency. Regions are boxed with coloured lines that indicate threshold values for the number of generations in which the _A1_D allele frequency was greater than 90%. The black lines on each axis indicate the default value for the parameter being varied. **Row 4 (j-l)**. Difference in the maximum _A_D allele frequency between population one and two. Regions boxed with a red line are simulations in which the maximum allele frequency of population one or two was at generation 100 and the outcomes may change if additional generations are simulated.

Like the generation of type-1 resistance alleles, Cas9:gRNA cut-rates that are less than saturating (<100%) have been reported for multiple HEGs [15, 18, 19, 27, 30, 33, 38]. In Fig 3 we show A allele dynamics of simulations with a cut-rate of 85%, with the allele dynamics of all loci shown in S10 Fig. The spread of the self-perpetuating drive is delayed, but otherwise largely unaffected by a lowering of the cut-rate as each element can simply try again in the next generation (Fig 3a). With a cut-rate of 85% the two DCDs reach maximum A element frequencies of 79.7% and 92% for the direct DCD and indirect DCD, respectively (Fig 3b and 3c). Interestingly, despite the lowered cut-rate, type-1 alleles are still produced in large numbers with the direct DCD design, even exceeding the drive allele frequency by the end of the simulation. Simulations of a range of cut-rates are presented in Fig 3d–3f and S11 Fig.

With a reduction in the cut-rate the DCD B element can now be passed along together with an uncut _A_T allele (as opposed to necessarily a drive element or a resistance allele when the cut-rate is 100%). This expands the possible genotypes under which phantom cutting can occur to include genotypes like _A_TT/_B_N^A^T and creates the possibility of simultaneous cleavage of homozygous target alleles at the A locus. This complicates the nuclease conversion scheme as HDR could, in principle, recursively regenerate a T allele from the homologous T allele, opening up the opportunity of repeated and simultaneous cutting events. The simulation presented in Fig 3 were performed under the assumption that HDR does not occur when a TT genotype is cleaved, instead, additional resistance alleles are generated. In S12 Fig we present simulations with various cut-rates assuming HDR is possible, and individual T alleles in a TT genotype are cut sequentially. Regardless of the approach taken, the indirect DCD is more invasive than direct DCD at equivalent cut-rates and produces fewer type-1 resistance alleles.

The most straightforward way to compensate for a reduction in invasiveness may be to increase the drive frequency at release. However, for the self-perpetuating drive, this has a very limited effect when the reduction in invasiveness is due to an extremely low cut-rate. Under these conditions (cut-rates lower than ≈15%) the drive element remains unable to overcome its fitness costs independent of the drive frequency at release (Fig 3g). In contrast, any reduction in cut-rate affects the DCD much earlier through the loss of co-inheritance of the different drive elements (Fig 3h and 3i). For these conditions, an increased release frequency can allow DCDs to reach frequencies where orphaned drive elements are likely to be reacquainted by random mating. For DCDs, the frequency-dependent consequence of a reduction in cut-rate limits the DCDs ability to invade population two when the cut-rate is reduced (S13 Fig). This frequency-dependent effect does not apply to the self-perpetuating drive as only a single-element needs to be inherited to affect a cutting reaction at the target locus. Fig 3j–3l shows this consequently provides a broad range of cut-rate and release frequency parameter combinations under which the DCDs spread to high frequencies in population one, but not population two.

The production of type-1 resistance alleles through phantom cutting from isolated B elements causes the direct DCD to be more severely affected by reductions in cut-rates compared to indirect DCD. In addition to failing to spread with low cut-rates, the type-1 resistance alleles produced by DCDs may persist and theoretically impact a future release of an otherwise appropriately tuned drive release targeting the same locus. Next, we set out to determine how deposition would affect DCDs and potentially reveal additional differences between the drive designs.

### Maternal deposition can aid daisy-chain containment

The inclusion of deposition greatly expands the genotypes that can be exposed to cutting by the drive. We assume exclusively maternal deposition without any paternal deposition, and deposition occurs uniformly into each progeny, with the deposition cut-rate determining the fraction of target alleles that are cut in each progeny. The most straightforward deposition scenario is the simultaneous deposition of the Cas9 protein and gRNA as a complex. However, gRNAs in CRISPR HEGs are generally expressed from (putatively) constitutive pol III promoters. In line with experimental data [27, 31, 38, 39], in our simulations embryonic cutting can also occur when the Cas9 protein is deposited into an individual that has inherited a gRNA expressing transgene from their father. In contrast, due to the reduced stability of the isolated gRNA [40–42], we assume that the pre-complexing of Cas9 and a gRNA in the mother is necessary for the gRNA to be deposited. Generally, cutting by the deposited nuclease is thought to occur much earlier in development, when HDR rates are much lower and HDR may in some cases be impossible due to physical isolation of homologous chromosomes [43–45]. As such, we assume that HDR repair does not occur in the early embryo and all cuts result in resistance alleles.

**Fig 4:**
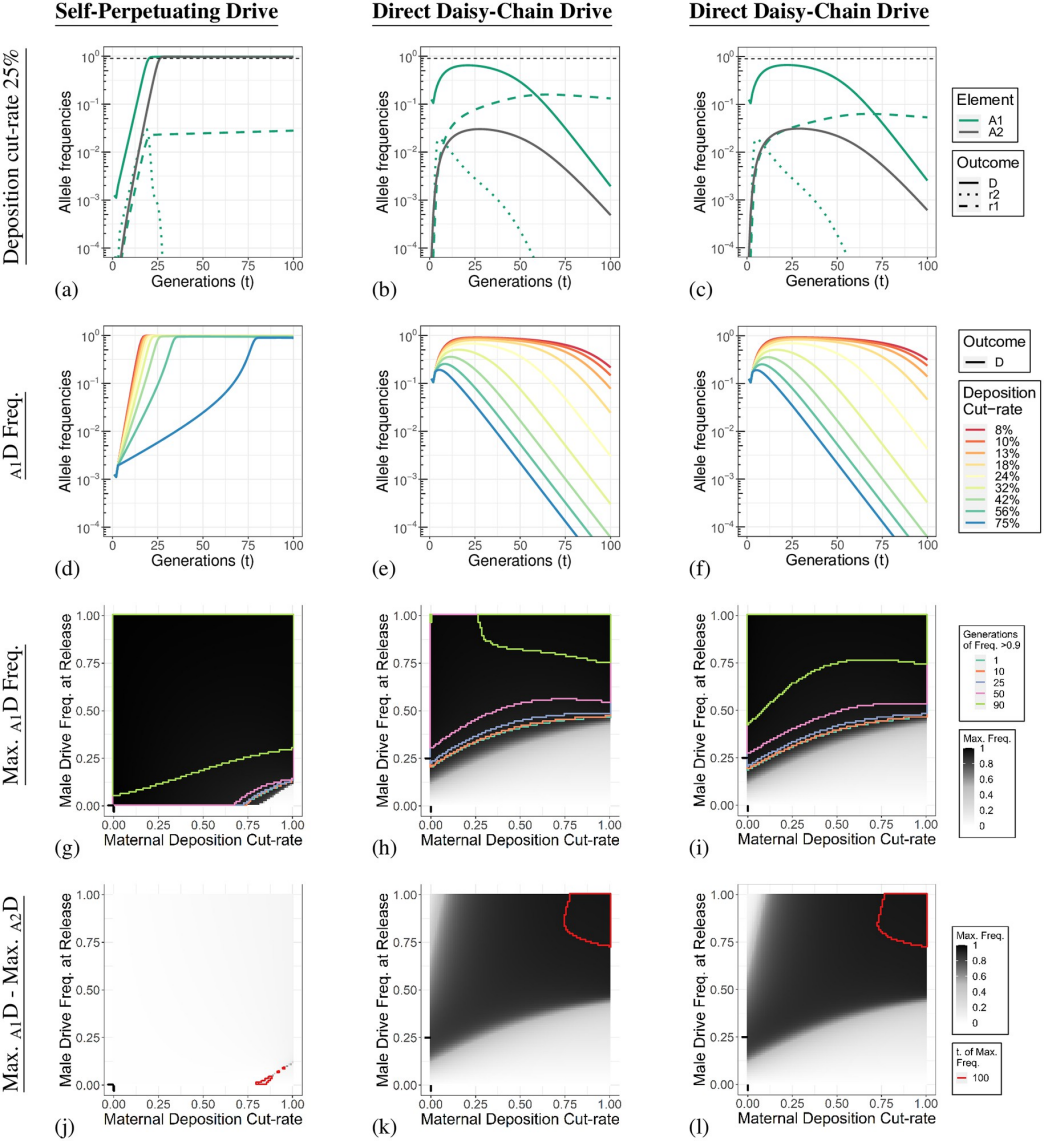
Maternal deposition affects the drives when they are at low frequencies. **Column 1**. Self-Perpetuating Drive. **Column 2**. Direct Daisy-Chain Drive. **Column 3**. Indirect Daisy-Chain Drive. **Row 1 (a-c)**. Individual allele dynamics with a maternal deposition cut-rate of 25%. The thin dashed line indicates a frequency of 90%. An allele at locus A in population one is indicated by _A1_ (green lines), and a drive allele at locus A in population two is indicated by _A2_ (gray line). Type 1 and 2 resistance alleles are not shown for population two in these panels. The allele dynamics for all loci are shown in S14 Fig. **Row 2 (d-f)**. A locus drive allele dynamics for population one with different maternal deposition cut-rates. Allele dynamics of the A locus drive allele in population two, and type-1 resistance alleles for both populations are shown in S15 Fig. **Row 3 (g-i)**. Parameter sweep for the male drive frequency at release and maternal deposition rates. The shading of the heat map indicates the maximum _A_D allele frequency in population one reached within the 100 generations simulated. The maximum frequency of _A_1 alleles and outcomes for population two are shown in S18 Fig. An immediate decline after release results in the maximum frequency being the release frequency. Regions are boxed with coloured lines that indicate threshold values for the number of generations in which the _A1_D allele frequency was greater than 90%. The black lines on each axis indicate the default value for the parameter being varied. **Row 4 (j-l)**. Difference in the maximum _A_D allele frequency between population one and two. Regions boxed with a red line are simulations in which the maximum allele frequency of population one or two was at generation 100 and the outcomes may change if additional generations are simulated.

In Fig 4a–4c we show the A locus allele dynamics of simulations with a maternal deposition cut-rate of 25%. In contrast to the outcomes when the cut-rate is changed, maternal deposition affects the two DCDs very similarly. The two DCDs reach maximum _A1_D element frequencies of 64.5% and 66.6% for the direct DCD and indirect DCD, respectively. For both DCDs, the B drive element carrying the nuclease drops in frequency rapidly due to its association with deposition-induced fitness costs at the A locus (S14 Fig).

In the previous simulations, the fitness cost of type-2 alleles were only experienced by those individuals that inherited it, not the individual in whose germline it was generated. To account for somatic genotype conversion by embryonic cutting, fitness costs are applied after maternal deposition-based gene conversion occurs (but before expression-based conversion in the germline). Any individual in which its _A_T alleles are cut due to deposition has its fitness lowered in proportion to the combined fitness of the individual genotypes of the now mosaic individual. Although cutting of _B_ T alleles can occur due to deposition, resistance mutations are neutral at this locus. However, deposition still reduces the proportion of uncut target alleles available for subsequent homing in the germline. It is important to note that despite the A drive element being a rescue, the drive inheriting individual are fully affected by resistance alleles that emerge from deposition as the target gene is haplolethal.

Fig 4d–4f show the _A1_D allele dynamics for different maternal deposition rates. The _A2_D and _A_1 allele dynamics are shown in S15 Fig. The same simulations were performed without the possibility of gRNA deposition. Restricting deposition in such a way may, on the face of it, be expected to aid the drives. However, it actually makes the fitness costs associated with deposition exclusively tied to inheritance of a gRNA expressing drive element, favouring individuals that do not inherit the gRNA expressing drive element. There may be more complex interactions with nuclease-only deposition for the two DCD designs; however, we find no substantial differences in the overall outcomes (S16 Fig).

There have been several reports of high rates of inheritance bias from maternally deposited nuclease (shadow drive) [27, 31, 38, 39]. This appears to be observed mainly in cases where Cas9 is provided by the mother and the gRNA gene from the father [8]. S17 Fig shows simulations of different maternal deposition rates with the possibility of shadow drive. We find that this specific scenario is so rare (and impossible for the self-perpetuating drive) that it has a negligible impact on the drive outcomes.

Finally, we tested how the drive frequency can be adapted to compensate for increasing rates of maternal deposition (Fig 4g–4i and S18 Fig). This shows that major changes in the deposition rate can be compensated for with relatively minor changes in the release frequency. This is in contrast to the same analysis described for changes to the germline-based cutting rate, which displayed a much more linear relationship. Strikingly, spread into population two was much more limited for DCD with maternal deposition, even at high release frequencies (S18 Fig). This provides a large parameter space with substantially higher drive spread into population one compared to population two (Fig 4j–4l) for the DCDs. The explanation for this is that once all target alleles have been cut, deposition ceases to have any consequence. However, at low frequencies, such as with the migration of drive-carrying individuals to a population of mostly wild-types, the deposition-induced fitness costs still occur and limit the drives ability to spread.

## Discussion

Here we have investigated the effect of drive element fitness, type-1 resistance alleles, cut-rate, and maternal deposition on two alternative daisy-chain gene drives (DCDs). The DCDs that we model are the direct DCD (_A_E/_B_N^A^/_C_G^B^), and indirect DCD (_A_G^A^/_B_N/_C_G^B^), each composed of three elements (downstream to upstream: A, B, C). In each case, we have contrasted our results with the behaviour of a self-perpetuating drive (_A_N^A^) for which more experimental and other computational results have been reported on the effect of fitness costs [20, 46], resistance alleles [20, 46, 47], cut-rate [20], and deposition [46, 48]. Despite using the same underlying inheritance biasing mechanism, we find that the performance of the DCDs can differ from a single-element self-perpetuating drive in important ways. However, there are important limitations to our model and the approach taken. Foremost among them is that we capture very little of the complexity of the life-history traits of the target species. Furthermore, our deterministic simulations of panmictic populations with simple migration will not adequately reflect any real-world populations. Yet, we expect that the broader relationships we have highlighted will remain true and can inform the design and optimisation of split and daisy-chain gene drives.

We find that the exponential nature of drive replication has different implications for the self-perpetuating drive and daisy-chain gene drives. In our simulations of replacement drives, the self-perpetuating drive generally has binary outcomes. When the drive can replicate itself faster than the drive is lost by fitness defects, the exponential replication of the drive will allow it to spread to high frequencies in both populations. If the drive is extremely inefficient, it will not spread at all. There is only a narrow set of parameters where the replication of the drive element is closely matched by the loss of drive elements, and the drive does spread but is substantially slowed. While the drive is spreading, more drive carriers are lost due to generational fitness effects, requiring more copying events (and therefore cutting) to reach equivalent frequencies compared to a faster spreading drive. The increased number of cutting events results in the generation of more resistance alleles. Whereas a self-perpetuating drive gains independence from the starting conditions and nuclease efficiencies from its sustained exponential spread, the daisy-chain gene drive is affected in the opposite way. The limited window of exponential replication experienced by DCDs causes their maximum frequency to be highly dependent on the starting conditions. Minor increases or decreases in efficiency will compound generation upon generation and can substantially alter the maximum frequency the drive can achieve, and its potential to spread beyond the target population. In our simulations, this compounding difference in efficiency is most apparent with changes to the drive allele fitness. However, type-1 resistance alleles, cut-rate, and maternal deposition also affect the balance of drive allele generation to loss and additionally have more specific interactions with the DCD design.

The spread of DCDs is substantially impacted by factors that can increase the effective linkage disequilibrium between different elements. Under ideal conditions, upstream daisy-chain elements are always inherited together with their downstream drive element (A for B and B for C) because those upstream elements cause the downstream element to switch from a heterozygous to homozygous state. However, reductions in cut-rate and the generation of resistance alleles in the germline or in the early embryo through deposition can cause upstream elements to be inherited without the corresponding downstream element. In addition to lowering the inheritance biasing rate, this also lowers the probability that in a subsequent generation, the DCD elements are in a configuration that allows for inheritance biasing to occur at all. For a self-perpetuating drive, all drive components are housed on the same element and cannot segregate away from each other.

The downside of linkage disequilibrium between daisy-chain elements can be further exacerbated by phantom cutting. This occurs with the direct daisy-chain B element (_B_N^A^), which can cut a wild-type A locus even when the B and A drive elements have segregated away from each other, generating additional resistance alleles. In our simulations, the production of additional dominant lethal type-2 resistance alleles at the A locus by phantom cutting, and the loss of B elements by association, has only a limited impact. This is because while an indirect DCD B element isolated from its A element cannot phantom cut, it also no longer contributes to the spread of the A element unless it is reunited with an A element by random mating. Reunification of B and A elements is only common if the drive is already at a high frequency, and by then additional B elements have been created, reducing the significance of the direct DCD B elements that have been lost. However, the effect of phantom cutting on the type-1 resistance allele frequency is more problematic. The additional production and biasing of type-1 resistance alleles by direct DCD phantom cutting substantially increases their frequency compared to the indirect DCD and self-perpetuating drive.

Throughout this study, we have exclusively discussed three-element DCDs. Incorporating additional DCD elements can increase invasiveness, as has been shown by modelling by others [9, 34]. This can be achieved by adding upstream gRNA elements (e.g., _D_G^C^), however, this now provides additional opportunities for phantom cutting (e.g., _B_N/_C_T/_D_G^C^). Upstream drive element may be expected to reach lower maximum frequencies than downstream elements, limiting the impact of resistance alleles generated by phantom cutting at these sites. Nonetheless, relatively complex additional modifications (e.g., orthogonal nucleases) would likely be required to avoid phantom cutting with longer DCDs.

The default release frequency was chosen to allow for high frequency spread of the two DCDs, and should be considered roughly tuned to our chosen drive efficiency and fidelity parameters. As a consequence, it is not a surprise that additional inefficiencies in the drive mechanism immediately affect the DCDs ability to approach fixation. Nevertheless, we show that the DCDs spread can be severely impacted by relatively minor changes to the drive efficiency. For a real-world release, the efficiency of the drive must be known to a high degree of precision to be able to tune a DCD release to the intended application. Relatively small errors in estimation or differences between laboratory and field conditions may allow the daisy-chain drive to spread substantially less or more than predicted. As we have shown, higher release frequencies can buffer the DCD against additional inefficiencies, but this may be undesirable if spread beyond the intended population is to be minimised. However, for many applications, there may be a large difference between the minimal release frequency needed and a release frequency that could allow for unacceptable spread beyond the target population. For those cases, the minimal required DCD release frequency can be safely exceeded to buffer against shortcomings in the drive efficiency. In addition, the risk of undesired additional spread by a DCD may also be mitigated by a slow ramp-up of release frequencies and continuous monitoring of spread. Nonetheless, our results indicate that high inheritance biasing efficiency and fidelity will be required for a DCD to achieve the design’s potential as useful middle ground between a self-perpetuating drive and non-drive inundative releases.

To date, inheritance biasing efficiency and fidelity exceeding our default parameters (cut-rate of 100%, HDR rate of 95%) has been achieved in *Anopheles gambiae* mosquitos in a single-element construction [49]. These results are encouraging for developing a DCD in the same species; however, it is unclear how the results with a single-element extend to a DCD. Multiple simultaneous DNA breaks have been reported to cause additional cellular stress [50] and interchromosomal abnormalities [51–55]. While some drives that bias multiple loci simultaneously have been reported [28, 56], to our knowledge, no multigenerational assessment of a DCD has been published. It remains to be demonstrated that a DCD can achieve similar efficiency and fidelity as a single-element drive. Furthermore, to date, drive efficiencies have been low in other potential target species such as *Aedes aegypti* [14, 29, 33] and *Mus musculus* [15, 18, 19], or high, but severely affected by maternal deposition, in *Anopheles stephensi* [25]. We expect that substantial improvements need to be made to allow HEGs in these species to function effectively with a DCD design.

It is important to note that there are many ways in which deposition can manifest (e.g., paternal vs. maternal deposition rates, deposition patterns for the gRNA, Cas9, and Cas9:gRNA complex, mRNA or protein deposition, and deposition rates from drive homozygous or heterozygous parents, repair rates through development) and many factors that influence the consequences of deposition (e.g., recessive and dominant fitness costs, life stage of fitness cost manifestation, threshold dependence of somatic conversion). As such, the deposition simulations performed here necessarily present a small subset of possible conditions and highlight that the consequence of deposition is context- and design-dependent. As more experimental data become available, further work can specifically test the deposition conditions observed for daisy-chain gene drives.

One possible way to address the increased nuclease efficiency and fidelity sensitivity we have found with DCDs is to tightly link individual elements by reducing their recombination distance. The elements should be close enough on the same chromosome, so segregation by meiotic recombination is rare but far enough that the homing events at each locus are still independent. This would negate the issues we find of premature dissociation of drive elements and phantom cutting; upstream elements should very rarely segregate away from downstream elements, even with low inheritance biasing efficiency and fidelity. However, there are a number of reports of drives designed to function through homing, also affecting the inheritance of a separate sequence located on the same or homologous chromosome [11, 14, 29, 31, 38, 57]. This collateral inheritance bias could, in a daisy-chain with tightly linked elements, cause two or more elements to bias themselves (more) like a self-perpetuating drive [7]. Daisy-chain drives in such a configuration may require higher safety assessments to ensure that it remains self-limiting in phased trials from the laboratory to the field.

An important consequence of DCD design that we have not considered in our simulations, that may be addressed in future work, is the potential interference between different drives. Cutting by the direct DCD A element is dependent on nuclease expression from another element for its function. However, the nuclease could be supplied by a different drive altogether. A rogue Cas9-based self-perpetuating drive could cause an indirect DCD drive A element to piggyback on the Cas9 expression and spread together with the self-perpetuating drive. This is similar to the behaviour of some reversal drives [58]. This piggybacking behaviour is not possible for the otherwise inferior direct DCD.

## Materials and methods

### Overview

The model we present here is capable of tracking the frequency of different genotypes through discrete life stages and generations. To facilitate that, we devised a nomenclature system capable of defining the range of possible genotypes and parental deposition states that individuals can take on for daisy-chain gene drives (the most complex case we consider). This is required to model a range of possible different drive designs and the many different individual states that a single drive can give rise to during its spread. During the embryonic and germline life stages, drive-induced genotype conversion occurs, changing the relative frequency of the genotypes. We use non-overlapping discrete-generation recursion equations for genotype frequencies, treating males and females separately. This method is applied in a simple deterministic allele frequency simulation of a single release of gene drive individuals into a population of all wildtypes.

### Stages and genotype nomenclature

The model tracks the frequency of different genotypes through different life stages. Each discrete generation (t), the same stages are repeated. The stages and their corresponding frequency vectors are: zygote (**z**), embryonic (**e**), germline (**g**), and gamete (**p**) stages. All vectors apart from the zygote vector are sex-specific, and male versions are indicated with a bar below the symbol (e.g., **p**). The overall genome frequency may vary through these stages; however, each generation the frequency is normalised to 1. For the particular drive system we are considering, d is the number of unique diploid genotypes, while h is the number of possible haploid genotypes. As such, **z**_i_ is the frequency of the i^th^ zygotic genotype, with i ranging from 1 to d.

Throughout each stage, the genomes maintain their genotype unless they are exposed to a gRNA:nuclease complex and contain a target allele (T) that can be cut. The other alleles we consider are type-1 (1), functional, and type-2 (2) non-functional resistance mutations and drive alleles. The symbol for the drive alleles is distinguished by whether that allele contains a nuclease (N), no nuclease but a gRNA (G), or neither (E). The N allele can carry a gRNA, but does not necessarily have to. None of the alleles except T can be cut but, if present, can influence the repair outcome of the T allele on the homologous chromosome. The drive elements can, depending on the specifics of the drive, provide the components necessary for a cut to occur at another locus but do not influence the repair outcomes at that other locus.

To model daisy-chain drives, we must further distinguish genotypes at different genomic loci. The defining feature of the persistence of inheritance bias for a daisy-chain element is its priority in the chain [9]. Sequentially going from A, B, C, etc. To aid in interpretation, each element’s genotype (diploid combinations of: T/N/G/1/2) has a letter subscript that denotes its order in the daisy-chain (e.g., _A_TT). Any genotype with a different letter subscript will be at a different genomic locus. For drive elements that carry a gRNA, a superscript indicates which T allele locus they target (e.g., _A_N^A^ or _C_G^B^). Table 1 lists the genotype notation of the drives studied in this publication and the two element split-drive.

**Table 1:** Genotype specification of drive designs. Drives are listed in their heterozygous state.

Genotype	Name
_A_N^A^T	Self-Perpetuating Drive
_A_G^A^T/_B_NT	Split Drive
_A_ET/_B_N^A^T/_C_N^B^T	Noble Daisy-Chain Drive
_A_ET/_B_N^A^T/_C_G^B^T	Direct Daisy-Chain Drive
_A_G^A^T/_B_NT/_C_G^B^T	Indirect Daisy-Chain Drive

### Haploid to diploid stage

Each discrete generation, the current genotype frequencies can change due to the action of the drive and fitness effects (S1(a) Fig). For generations 2–100, this process starts by the combining the haploid genotype frequencies of the previous generation from the male (**p**^*t*−1^) and female (**p**^*t*−1^) individuals to form the frequency vector of the zygotes (**z**^*t*^) (Eq 1). For generations 2–100, there is a single vector **z**, since, in our model, sex effects manifest only at the embryonic stage. However, for the first generation, the embryonic frequency is calculated separately for males and females according to the release frequencies. As such, released individuals are reduced according to their fitness before their opportunity to mate.

The model tracks the deposition of drive components from one generation to another. For Cas9 deposition, we differentiate each allele received by the zygote by a sex-specific Cas9 deposition factor (_0_ or _1_). We do not differentiate whether the deposition comes from a nuclease element heterozygote, or homozygote parent. For example, a haploid T}_0_ allele has come from a non-Cas9 carrying diploid parent (e.g., TT), while a T}_1_ must have come from a nuclease carrying parent (e.g., TN). In the diploid genomes these notations are combined, with the drive status of the male parent listed first (e.g., }_0*_) and then female parent (e.g., }_*0_). Eq 2 illustrates the combining of **p**^*t*−1^ and **p**^*t*−1^ to form **z**^*t*^. Note, that many genotypes are functionally equivalent and will be summed when reporting the outcomes of the drive (e.g., {T1}_00_ = {1T}_00_, {**p**_*i*_/**p**_*j*_}_00_ = {**p**_*j*_/**p**_*i*_}_00_, {**p**_*i*_/**p**_*j*_}_11_ = {**p**_*j*_/**p**_*i*_}_11_, {**p**_*i*_/**p**_*j*_}_10_ ≠ {**p**_*j*_/**p**_*i*_}_01_). Deposition of the gRNA is tracked in a similar way. The haploid genotypes are differentiated by the gRNAs that have been expressed in the diploid parent, and this is used in the calculation of deposition-based cutting in the diploid embryos.

$$\begin{array}{c} z^{t}={\underline{p}}^{t-1}\cdot p^{t-1} \end{array}$$ (1)

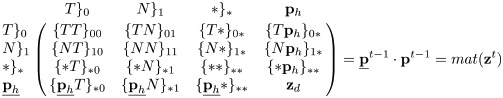
(2)

### Fitness costs, and genotype inter-conversion by deposition and expression

In the transition from the zygotic to the embryonic state, the model takes into account deposition and fitness effects. Through deposition, each individual zygotic genotype (**z**_*i*_) can give rise to multiple embryonic genotypes (**e**). Genotype conversion is mediated by matrix **K**, which converts genotypes according to the deposition cut and repair rates. Furthermore, based on the fitness parameters (Fig 1c), we can, for each genotype, define a particular fitness cost (0–1) in the vector *θ*. The fitness cost vector has a female (*θ*) and male (*θ*) specific version because for some simulations sex-specific fitness costs are applied. The fitness of a particular genotype (*θ*_*i*_) is determined by multiplying the fitness costs of individual alleles. The dominant fitness cost imposed by a particular allele is applied only once, even if two copies are present. Some simulations impose an additional recessive fitness cost on the A locus, which only occurs if two loss of function alleles are present at A (e.g., _A_EE, _A_E2, _A_22). As the multiple genotypes produced by deposition are within the same mosaic organism, the fitness cost contribution of individual genotypes is summed and applied uniformly over all genotypes arising from the same zygotic genotype. The deposition conversion outcomes and fitness costs of any one genotype do not vary throughout a simulation, only the relative frequency of the input genotypes in the form of **z**. As such, conversion matrix **D**/**D** can be constructed that mediates both deposition conversion (**K**), and fitness costs (*θ*_*i*_ and *θ*_*i*_). Matrix **D** is the product of the row-wise multiplication of matrix **K**_*i*,*_ by *θ* and normalisation. When **z** is then subjected to conversion matrix **D** it produces the embryonic frequency vector **e** (Eq 3). Vector **e** represents the genotype frequency at the end of the embryonic stage, after deposition and fitness costs have been applied. Vector **e**/**e** is subsequently multiplied by the expression-based gene conversion matrix **C**/**C** to produce the gamete frequency vector **g**/**g** (Eq 4). The construction of matrices **C** and **K** is discussed after gamete production.

$$\begin{array}{cc} e^{t}=z^{t}\cdot D & \;{\underline{e}}^{t}=z^{t}\cdot \underline{D} \end{array}$$ (3)

$$\begin{array}{cc} g^{t}=e^{t}\cdot C & \;{\underline{g}}^{t}={\underline{e}}^{t}\cdot \underline{C} \end{array}$$ (4)

### Gamete production

The last step in each generation is the conversion of diploid genomes to haploid ones. This is done by multiplying the diploid genome frequency vector **g** by matrix **H** to form the haploid genome frequency vector **p** (Eq 5). Matrix **H** simply splits each diploid genome into the possible haploid genomes it can produce, an example of which is shown in Eq 6. An important step in this process is to keep track of nuclease and gRNA deposition. The presence of at least one N allele in the diploid germline genome causes the haploid genome to have an associated deposition marker 1 (*}_1_). If no N allele is present in the diploid genome, the deposition marker is 0 (*}_0_). Separately, any gRNA expressing elements in the diploid parent causes each haploid genome to carry an indicator for deposition of that gRNA (as long as Cas9 is deposited simultaneously). Like Cas9 deposition, this occurs even if the gRNA-expressing element is not inherited. We assume that alleles at separate loci segregate fully independently, as is shown in Eq 7 which shows **H** if two elements are considered.

$$\begin{array}{cc} p^{t}=g^{t}\cdot H & \;{\underline{p}}^{t}={\underline{g}}^{t}\cdot H \end{array}$$ (5)

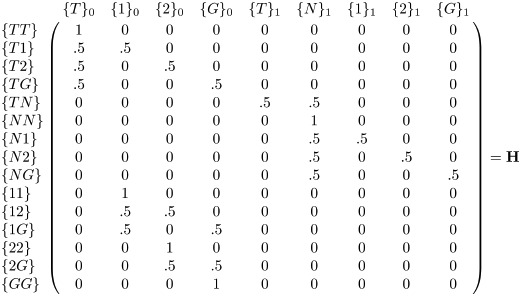
(6)

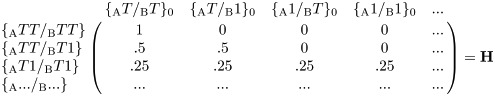
(7)

### Deposition and drive induced conversion

To model drive activity within an individual, we approach gene editing as the conversion of genomes from one genotypic state to another genotypic state. The probabilities underlying this will depend on the probability that a DNA cut will occur (*κ*) and the HDR rate (*α*), together with the ratio between type-1 and type-2 resistance mutations (*β*). From this, DNA repair can result in three outcomes: interchromosomal-homology-directed repair (*α*), type-1 resistance mutations ((1− *α*) ⋅ *β* = *μ*), and type-2 resistance mutations ((1− *α*) ⋅ (1 − *β*) = *ν*). The distinction between type-1 and type-2 mutations is dependent on the target locus, and in our model only relevant for accounting for fitness effects. The probability of all repair outcomes sums to 1. For each simulation, the value of each of these parameters is fixed and the default values are listed in Fig 1c. Each of the previous parameters is defined separately for maternal deposition-based conversion, which is indicated by _01_ (*κ*_01_, *α*_01_, and *β*_01_).

In our model, there are two stages where the drive can induce a genotype conversion: embryonic and germline. For each genotype and its associated deposition state, we define a rate at which it converts to the other possible genotypes or remains the same (the overall rate is always 1). These conversion probabilities are mediated by matrix **C** for the drive expression in the germline, and **K** for drive deposition in the embryo. For the majority of genotypes, the conversion rate maintaining the original genotype will be 1, and the conversion rate to all other genotypes will be 0. This is because only a select few genotypes can convert. The requirements for conversion are: first, the genotype must have a genomically expressed gRNA or deposited gRNA (in complex with Cas9). Second, that gRNA must target a locus that in that particular individual has one or more T alleles present. Lastly, there must be genomically expressed or deposited nuclease. If these conditions are met, we use the cut and repair probabilities to determine the conversion rate. If a T allele can be cut in the germline, *κ* ⋅ *α* of the T alleles will convert to whatever allele is on the homologous chromosome. *κ* ⋅ *μ* of the T alleles will convert to a type-1 resistance allele and *κ* ⋅ *ν* of the T alleles will convert to a type-2 resistance allele. Finally, 1-*κ* of the T alleles will remain uncut. These conversion rates populate matrix **C**, as shown in Eq 8. The exact same relationships hold for matrix **K** shown in Eq 9, with the *κ*_01_, *α*_01_, *μ*_01_, and *ν*_01_ parameters (with *α*_01_ being 0, except in the case of the shadow drive simulations). The conversion matrix for genotypes with TT is more complex. For a TT genotype to be cut, the nuclease and gRNA must be expressed from a separate locus, or alternatively the nuclease, or nuclease and gRNA can be provided by maternal deposition. For TT genotypes, we assume that *α* = 0, or in a separate set of supplemental simulations that alleles are cut one by one, resulting in the conversion probabilities shown in Eq 10.

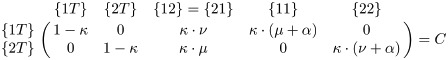
(8)

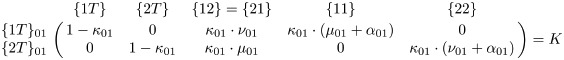
(9)

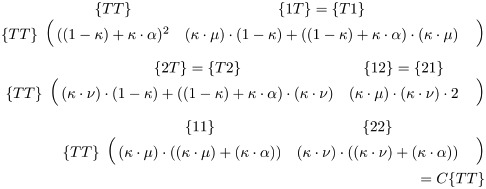
(10)

## Supporting information

S1 Fig Overview of the genotype conversion process that repeats each generation. **(a)** Illustration of the different stages of the model. **(b)** Names of the symbols used in a. Each genotype conversion step is expanded upon in the methods.
(TIF)

S2 Fig Individual allele dynamics with all parameters at default. **Row 1 (a-c)**. Individual allele dynamics for population one. **Row 2 (d-f)**. Individual allele dynamics for population two. **Column 1**. Self-Perpetuating Drive. **Column 2**. Direct Daisy-Chain Drive. **Column 3**. Indirect Daisy-Chain Drive. The thin dashed line indicates a frequency of 90%.
(TIF)

S3 Fig Phantom cutting occurs when an upstream element attempts to bias the inheritance of a downstream drive element that is not present. With cut-rates less than 100%, downstream T alleles can be inherited with a drive element that can allow the phantom cutting of a homozygous TT genotype.
(TIF)

S4 Fig Germline HDR rate parameter sweep. **Row 1 (a-c)**. Allele dynamics of the A locus drive element in population one. **Row 2 (d-f)**. Allele dynamics of the A locus drive element in population two. **Row 3 (g-i)**. Allele dynamics of the A locus type-1 resistance mutations in population one. **Row 4 (j-l)**. Allele dynamics of the A locus type-1 resistance mutations in population two. **Column 1**. Self-Perpetuating Drive. **Column 2**. Direct Daisy-Chain Drive. **Column 3**. Indirect Daisy-Chain Drive.
(TIF)

S5 Fig Migration rate parameter sweep. **Row 1 (a-c)**. Allele dynamics of the A locus drive element in population one. **Row 2 (d-f)**. Allele dynamics of the A locus drive element in population two. **Row 3 (g-i)**. Allele dynamics of the A locus type-1 resistance mutations in population one. **Row 4 (j-l)**. Allele dynamics of the A locus type-1 resistance mutations in population two. **Column 1**. Self-Perpetuating Drive. **Column 2**. Direct Daisy-Chain Drive. **Column 3**. Indirect Daisy-Chain Drive. Migration rates are 0.5^n^, with n from 1 to 8.
(TIF)

S6 Fig Simulation of different drive designs using previously published parameters. **Row 1 (a-d)**. Illustration of the daisy-chain design. The remaining rows are simulations of each daisy-chain drive under different release and HDR rates used by Noble et al. [9] ‘Fig.2B’. **Row 2 (e-h)**. Preset 1 is a simulation with a release frequency of 2% and HDR rate of 95%. **Row 3 (i-l)**. Preset 2 is a simulation with a release frequency of 2% and HDR rate of 60%. **Row 4 (m-p)**. Preset 3 is a simulation with a release frequency of 15% and an HDR rate of 60%. For all simulations, the allele fitness is: _A_D = 92%, _A_2 = 0%, _B_D and _C_D = 99.99%, _B_2 = 100%. Cut-rate = 100%. These simulations were of a single population. **Column 1**. Direct Daisy-Chain Drive. **Column 2**. Indirect Daisy-Chain Drive. **Column 3**. Direct Daisy-Chain Drive with Cas9 also expressed from the C drive element. **Column 4**. Indirect Daisy-Chain Drive with Cas9 also expressed from the C drive element. The thin dashed line indicates a frequency of 90%.
(TIF)

S7 Fig Simultaneous changes to the recessive and dominant fitness cost of the _A_D allele. **Row 1 (a-c)**. Maximum _A_D allele frequency in population two. **Row 2 (d-f)**. Maximum _A_1 allele frequency in population one. **Row 3 (g-i)**. Maximum _A_1 allele frequency in population two. The maximum frequency of _A_D in population one and the difference between population one and two is shown in Fig 2.
(TIF)

S8 Fig Male release frequency parameter sweep with a recessive lethal A drive element. **Row 1 (a-c)**. Allele dynamics of the A locus drive element in population one. **Row 2 (d-f)**. Allele dynamics of the A locus drive element in population two. **Row 3 (g-i)**. Allele dynamics of the A locus type-1 resistance mutations in population one. **Row 4 (j-l)**. Allele dynamics of the A locus type-1 resistance mutations in population two. **Column 1**. Self-Perpetuating Drive. **Column 2**. Direct Daisy-Chain Drive. **Column 3**. Indirect Daisy-Chain Drive. A heterozygous release frequency of 100% means that all males in population one are heterozygous drive carriers at the start of the simulation. This equates to a drive allele frequency of 50% among males and a frequency of 25% among all alleles when females are included (the y-axis value shown).
(TIF)

S9 Fig Male release frequency parameter sweep with a female-specific recessive lethal A drive element. **Row 1 (a-c)**. Allele dynamics of the A locus drive element in population one. **Row 2 (d-f)**. Allele dynamics of the A locus drive element in population two. **Row 3 (g-i)**. Allele dynamics of the A locus type-1 resistance mutations in population one. **Row 4 (j-l)**. Allele dynamics of the A locus type-1 resistance mutations in population two. **Column 1**. Self-Perpetuating Drive. **Column 2**. Direct Daisy-Chain Drive. **Column 3**. Indirect Daisy-Chain Drive. A heterozygous release frequency of 100% means that all males in population one are heterozygous drive carriers at the start of the simulation. This equates to a drive allele frequency of 50% among males and a frequency of 25% among all alleles when females are included (the y-axis value shown).
(TIF)

S10 Fig Individual allele dynamics with a germline cut-rate of 85%. **Row 1 (a-c)**. Individual allele dynamics for population one. **Row 2 (d-f)**. Individual allele dynamics for population two. **Column 1**. Self-Perpetuating Drive. **Column 2**. Direct Daisy-Chain Drive. **Column 3**. Indirect Daisy-Chain Drive. The thin dashed line indicates a frequency of 90%.
(TIF)

S11 Fig Germline cut-rate parameter sweep with no HDR repair of cut TT genotypes. **Row 1 (a-c)**. Allele dynamics of the drive element at the A locus in population two. **Row 2 (d-f)**. Allele dynamics of type-1 resistance mutations at the A locus in population one. **Row 3 (g-i)**. Allele dynamics of type-1 resistance mutations at the A locus in population two. Allele dynamics of the drive element at the A locus in population one are shown in Fig 3d–3f. **Column 1**. Self-Perpetuating Drive. **Column 2**. Direct Daisy-Chain Drive. **Column 3**. Indirect Daisy-Chain Drive.
(TIF)

S12 Fig Germline cut-rate parameter sweep with sequential cutting of TT genotypes. **Row 1 (a-c)**. Allele dynamics of the A locus drive element in population one. **Row 2 (d-f)**. Allele dynamics of the A locus drive element in population two. **Row 3 (g-i)**. Allele dynamics of the A locus type-1 resistance mutations in population one. **Row 4 (j-l)**. Allele dynamics of the A locus type-1 resistance mutations in population two. **Column 1**. Self-Perpetuating Drive. **Column 2**. Direct Daisy-Chain Drive. **Column 3**. Indirect Daisy-Chain Drive.
(TIF)

S13 Fig Simultaneous changes to the germline cut-rate and male drive carrier frequency at release. **Row 1 (a-c)**. Maximum _A_D allele frequency in population two. **Row 2 (d-f)**. Maximum _A_1 allele frequency in population one. **Row 3 (g-i)**. Maximum _A_1 allele frequency in population two. The maximum frequency of _A_D in population one and the difference between population one and two is shown in Fig 3.
(TIF)

S14 Fig Individual allele dynamics with a maternal deposition cut-rate of 25%. **Row 1 (a-c)**. Individual allele dynamics for population one. **Row 2 (d-f)**. Individual allele dynamics for population two. **Column 1**. Self-Perpetuating Drive. **Column 2**. Direct Daisy-Chain Drive. **Column 3**. Indirect Daisy-Chain Drive. HDR does not occur with deposition-mediated cutting, and all cuts result in resistance mutations following the 1:9 ratio of type-1 to type-2. The thin dashed line indicates a frequency of 90%. The dynamics of the direct and indirect DCD _B1_D elements may be more similar than initially expected from the genetics. However, when the DCD B elements are together with a drive element at A, there is no difference in deposition between the direct (_A_E/_B_N^A^) and indirect (_A_G^A^/_B_N) DCDs. Only when the B element is isolated from the A drive element does deposition affect the two designs differently. This only occurs when the B element segregates away with a type-1 resistance allele (100% expression based cut-rate, and type-2 resistance alleles are lethal). Although the resistance allele rate is substantially higher with deposition, isolation of _B_D from _A_D is still a rare event. Moreover, due to the high _A_D frequency, isolated B elements are rapidly reacquainted with a A drive element. Isolated _B_D elements are much less likely to be reacquainted with a _A_D element in population two, which is reflected in a more pronounced difference in _B_D allele dynamics between the indirect and direct DCDs.
(TIF)

S15 Fig Maternal deposition cut-rate parameter sweep. **Row 1 (a-c)**. Allele dynamics of the drive element at the A locus in population two. **Row 2 (d-f)**. Allele dynamics of type-1 resistance mutations at the A locus in population one. **Row 3 (g-i)**. Allele dynamics of type-1 resistance mutations at the A locus in population two. Allele dynamics of the drive element at the A locus in population one are shown in Fig 4d–4f. **Column 1**. Self-Perpetuating Drive. **Column 2**. Direct Daisy-Chain Drive. **Column 3**. Indirect Daisy-Chain Drive. HDR does not occur with deposition-mediated cutting, and all cuts result in resistance mutations following the 1:9 ratio of type-1 to type-2.
(TIF)

S16 Fig Maternal deposition cut-rate parameter sweep with no gRNA deposition. In these simulations only the Cas9 protein is deposited. **Row 1 (a-c)**. Allele dynamics of the A locus drive element in population one. **Row 2 (d-f)**. Allele dynamics of the A locus drive element in population two. **Row 3 (g-i)**. Allele dynamics of the A locus type-1 resistance mutations in population one. **Row 4 (j-l)**. Allele dynamics of the A locus type-1 resistance mutations in population two. **Column 1**. Self-Perpetuating Drive. **Column 2**. Direct Daisy-Chain Drive. **Column 3**. Indirect Daisy-Chain Drive. HDR does not occur with deposition-mediated cutting, and all cuts result in resistance mutations following the 1:9 ratio of type-1 to type-2.
(TIF)

S17 Fig Maternal deposition cut-rate parameter sweep with shadow drive. In these simulations, when the deposited Cas9 protein is paired with an expressed gRNA, DNA repair uses the germline HDR rate. If Cas9 and a gRNA are deposited simultaneously, this takes precedence, and HDR repair is not possible. In practise, shadow drive occurs only when the mother carries the Cas9 and the father provides the gRNA gene. Note that this scenario cannot occur with the self-perpetuating drive as the Cas9 and gRNA gene are always linked. **Row 1 (a-c)**. Allele dynamics of the A locus drive element in population one. **Row 2 (d-f)**. Allele dynamics of the A locus drive element in population two. **Row 3 (g-i)**. Allele dynamics of the A locus type-1 resistance mutations in population one. **Row 4 (j-l)**. Allele dynamics of the A locus type-1 resistance mutations in population two. **Column 1**. Self-Perpetuating Drive. **Column 2**. Direct Daisy-Chain Drive. **Column 3**. Indirect Daisy-Chain Drive.
(TIF)

S18 Fig Simultaneous changes to the maternal deposition cut-rate and male drive carrier frequency at release. **Row 1 (a-c)**. Maximum _A_D allele frequency in population two. **Row 2 (d-f)**. Maximum _A_1 allele frequency in population one. **Row 3 (g-i)**. Maximum _A_1 allele frequency in population two. The maximum frequency of _A_D in population one and the difference between population one and two is shown in Fig 4. HDR does not occur with deposition-mediated cutting, and all cuts result in resistance mutations following the 1:9 ratio of type-1 to type-2.
(TIF)
