# Supplementary figures and images for: Daisy-chain gene drives: The role of low cut-rate, resistance mutations, and maternal deposition
Source: PLoS Genet. 2022 Sep 19;18(9):e1010370. doi: 10.1371/journal.pgen.1010370 (PMC9521892; doi:10.1371/journal.pgen.1010370)

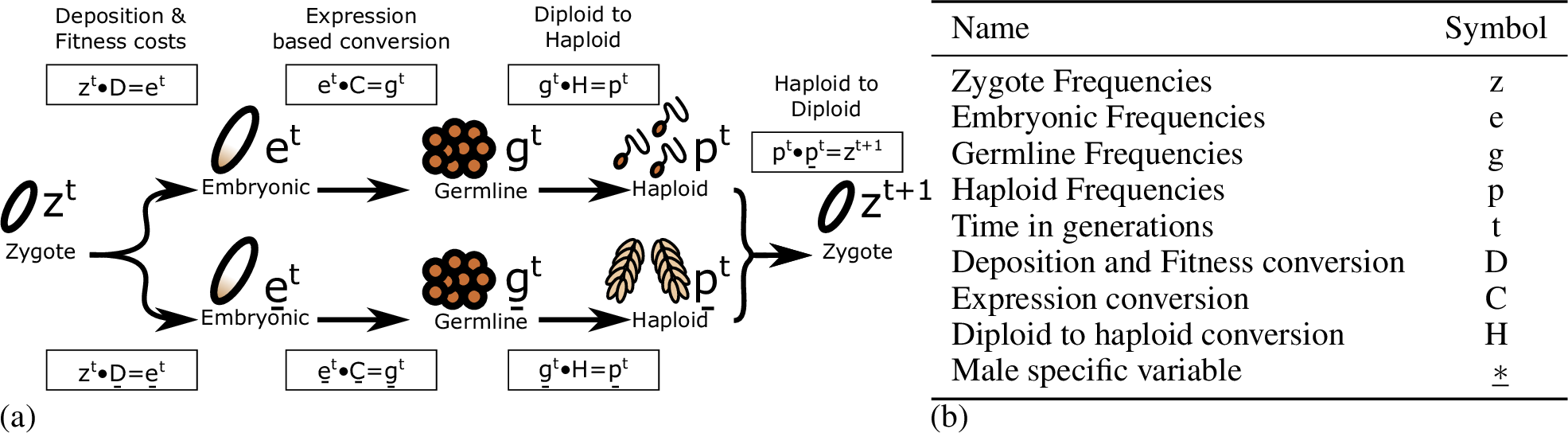

Supplement: S1 Fig — (a) Illustration of the different stages of the model. (b) Names of the symbols used in a. Each genotype conversion step is expanded upon in the methods. (TIF) [file pgen.1010370.s001.tif]

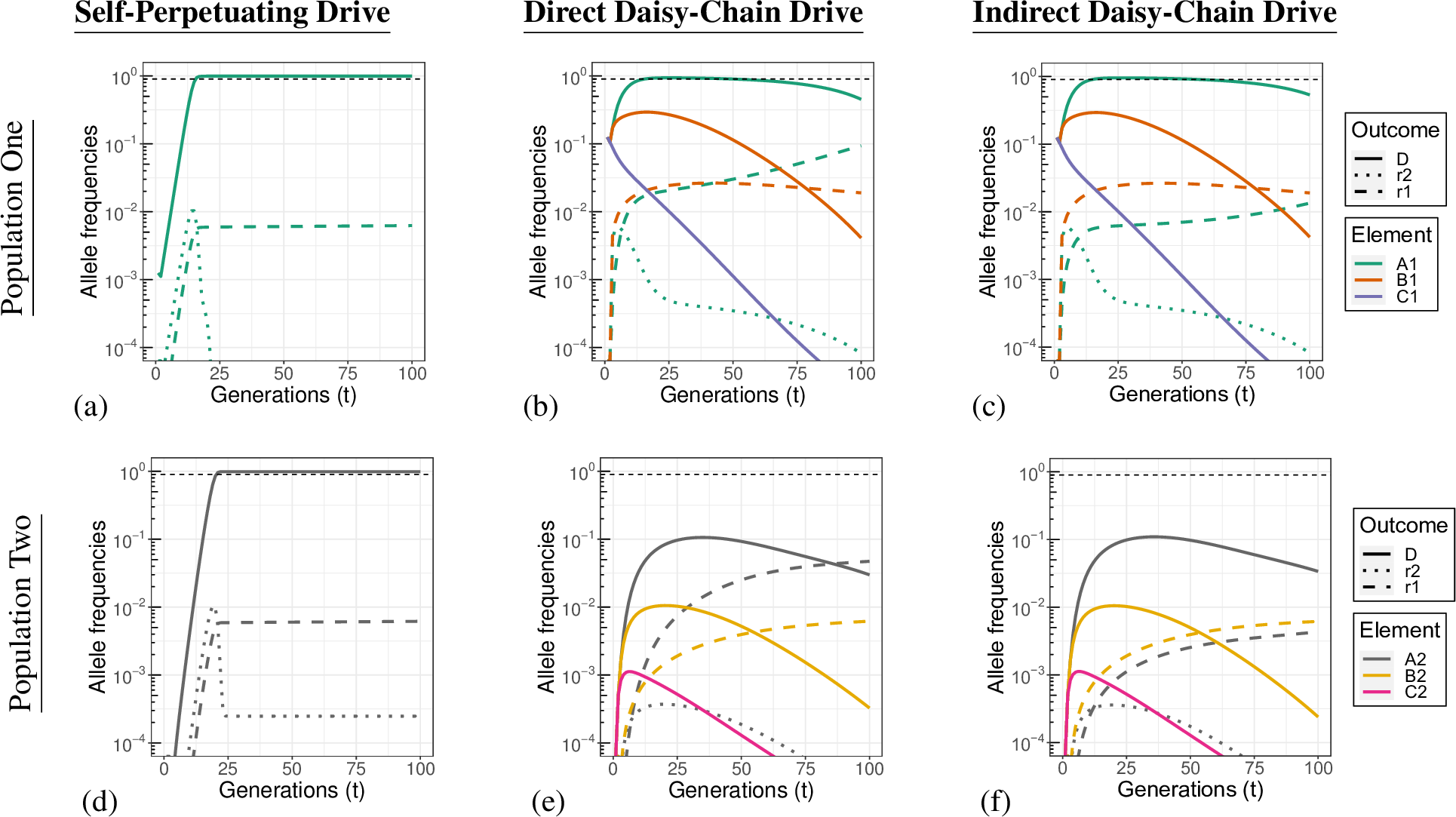

Supplement: S2 Fig — Row 1 (a-c). Individual allele dynamics for population one. Row 2 (d-f). Individual allele dynamics for population two. Column 1. Self-Perpetuating Drive. Column 2. Direct Daisy-Chain Drive. Column 3. Indirect Daisy-Chain Drive. The thin dashed line indicates a frequency of 90%. (TIF) [file pgen.1010370.s002.tif]

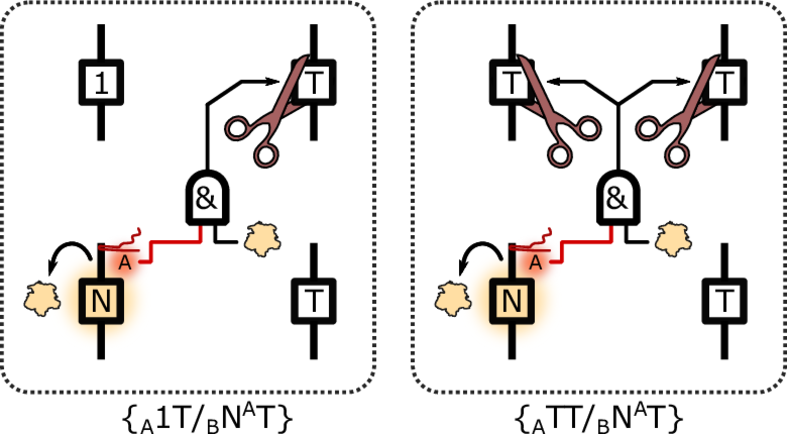

Supplement: S3 Fig — With cut-rates less than 100%, downstream T alleles can be inherited with a drive element that can allow the phantom cutting of a homozygous TT genotype. (TIF) [file pgen.1010370.s003.tif]

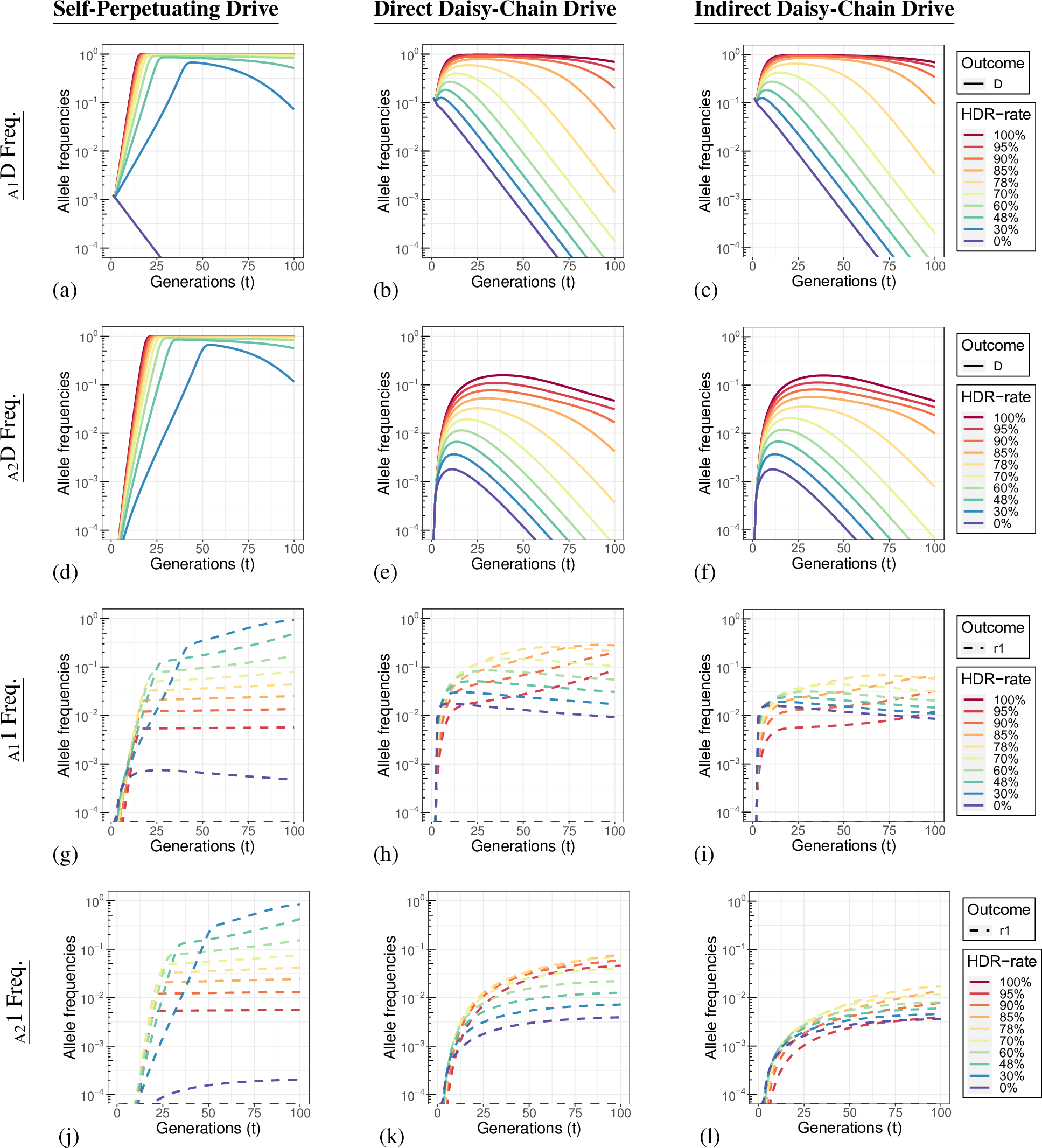

Supplement: S4 Fig — Row 1 (a-c). Allele dynamics of the A locus drive element in population one. Row 2 (d-f). Allele dynamics of the A locus drive element in population two. Row 3 (g-i). Allele dynamics of the A locus type-1 resistance mutations in population one. Row 4 (j-l). Allele dynamics of the A locus type-1 resistance mutations in population two. Column 1. Self-Perpetuating Drive. Column 2. Direct Daisy-Chain Drive. Column 3. Indirect Daisy-Chain Drive. (TIF) [file pgen.1010370.s004.tif]

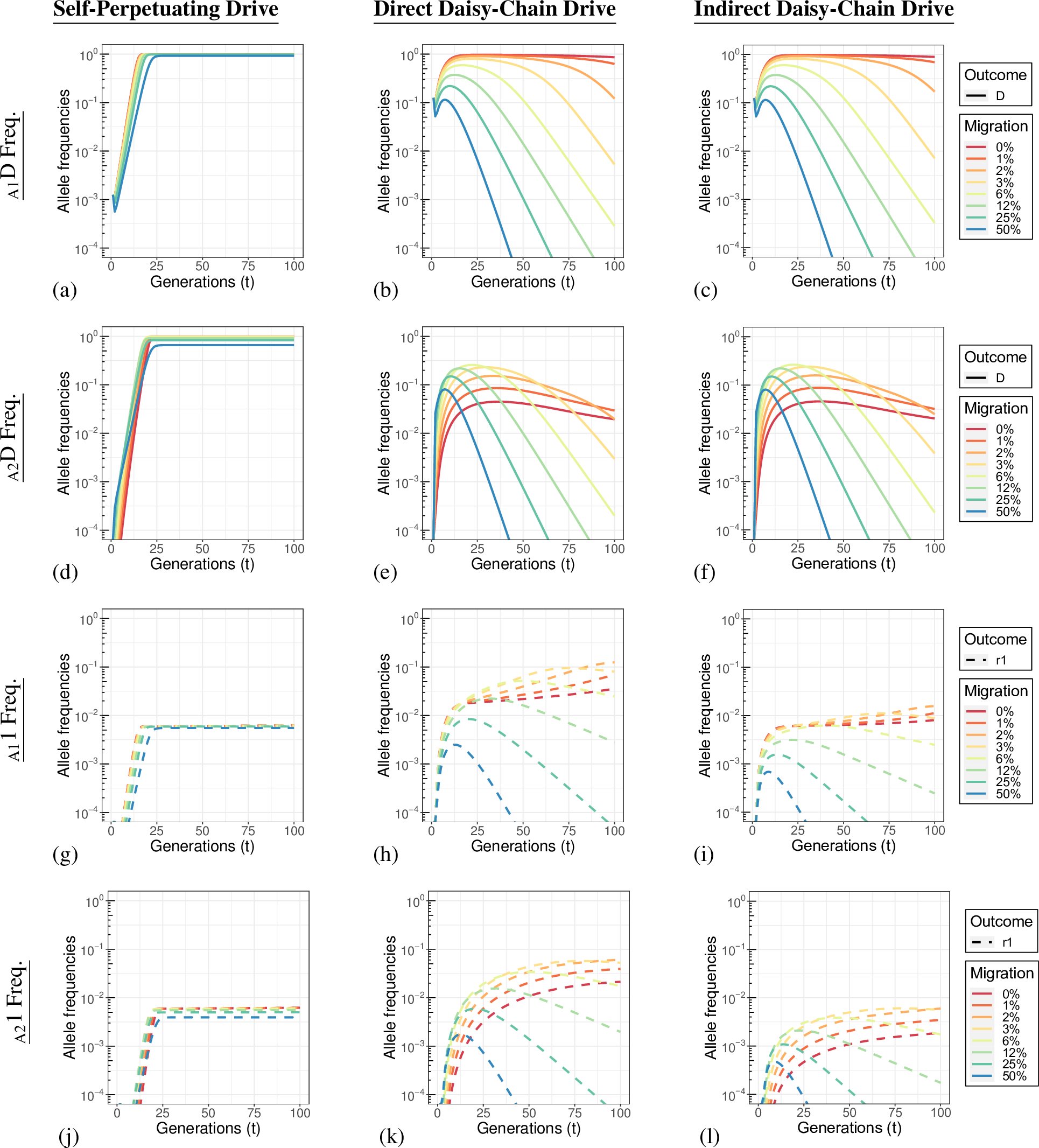

Supplement: S5 Fig — Row 1 (a-c). Allele dynamics of the A locus drive element in population one. Row 2 (d-f). Allele dynamics of the A locus drive element in population two. Row 3 (g-i). Allele dynamics of the A locus type-1 resistance mutations in population one. Row 4 (j-l). Allele dynamics of the A locus type-1 resistance mutations in population two. Column 1. Self-Perpetuating Drive. Column 2. Direct Daisy-Chain Drive. Column 3. Indirect Daisy-Chain Drive. Migration rates are 0.5n, with n from 1 to 8. (TIF) [file pgen.1010370.s005.tif]

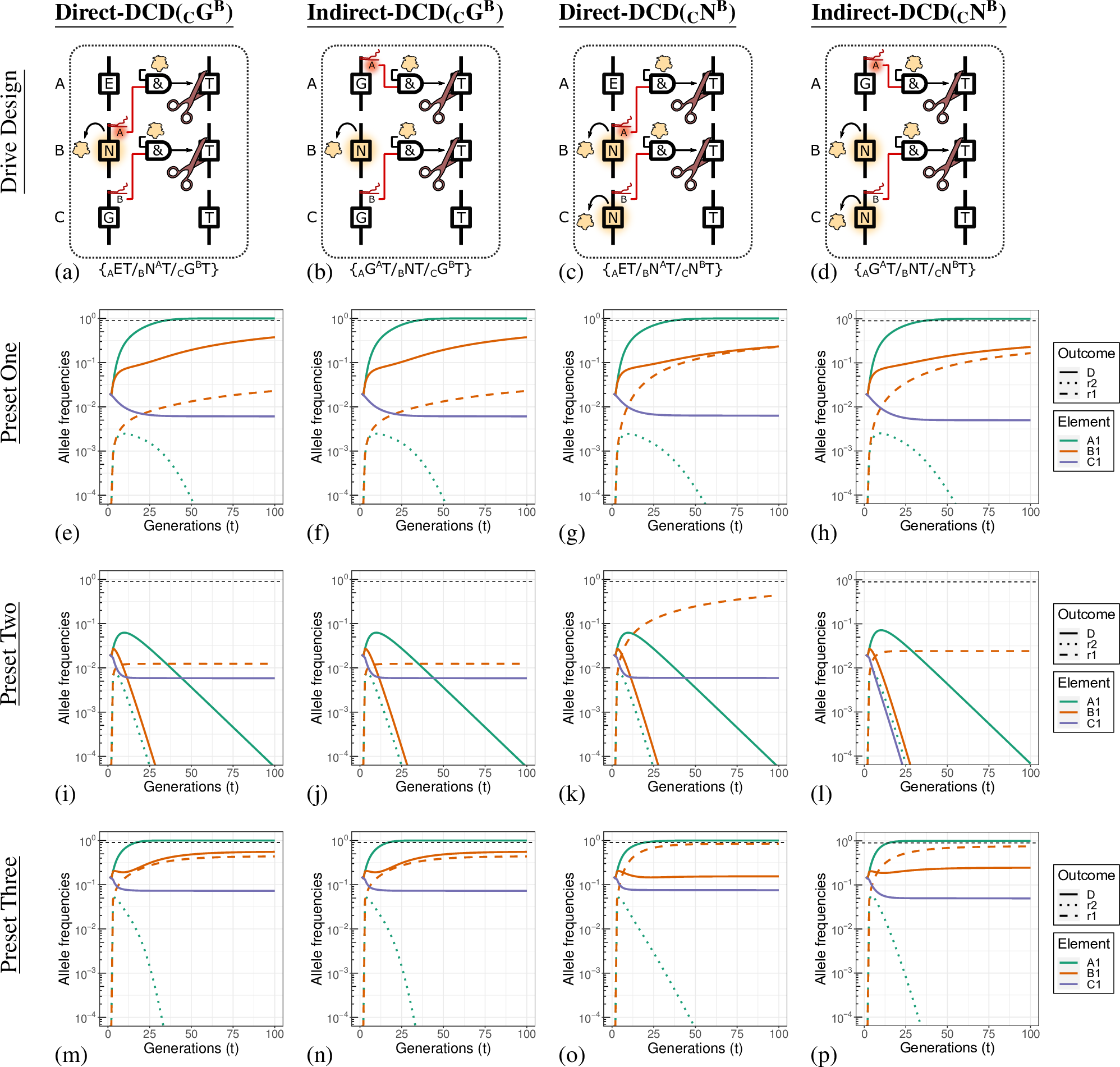

Supplement: S6 Fig — Row 1 (a-d). Illustration of the daisy-chain design. The remaining rows are simulations of each daisy-chain drive under different release and HDR rates used by Noble et al. [9] ‘Fig.2B’. Row 2 (e-h). Preset 1 is a simulation with a release frequency of 2% and HDR rate of 95%. Row 3 (i-l). Preset 2 is a simulation with a release frequency of 2% and HDR rate of 60%. Row 4 (m-p). Preset 3 is a simulation with a release frequency of 15% and an HDR rate of 60%. For all simulations, the allele fitness is: AD = 92%, A2 = 0%, BD and CD = 99.99%, B2 = 100%. Cut-rate = 100%. These simulations were of a single population. Column 1. Direct Daisy-Chain Drive. Column 2. Indirect Daisy-Chain Drive. Column 3. Direct Daisy-Chain Drive with Cas9 also expressed from the C drive element. Column 4. Indirect Daisy-Chain Drive with Cas9 also expressed from the C drive element. The thin dashed line indicates a frequency of 90%. (TIF) [file pgen.1010370.s006.tif]

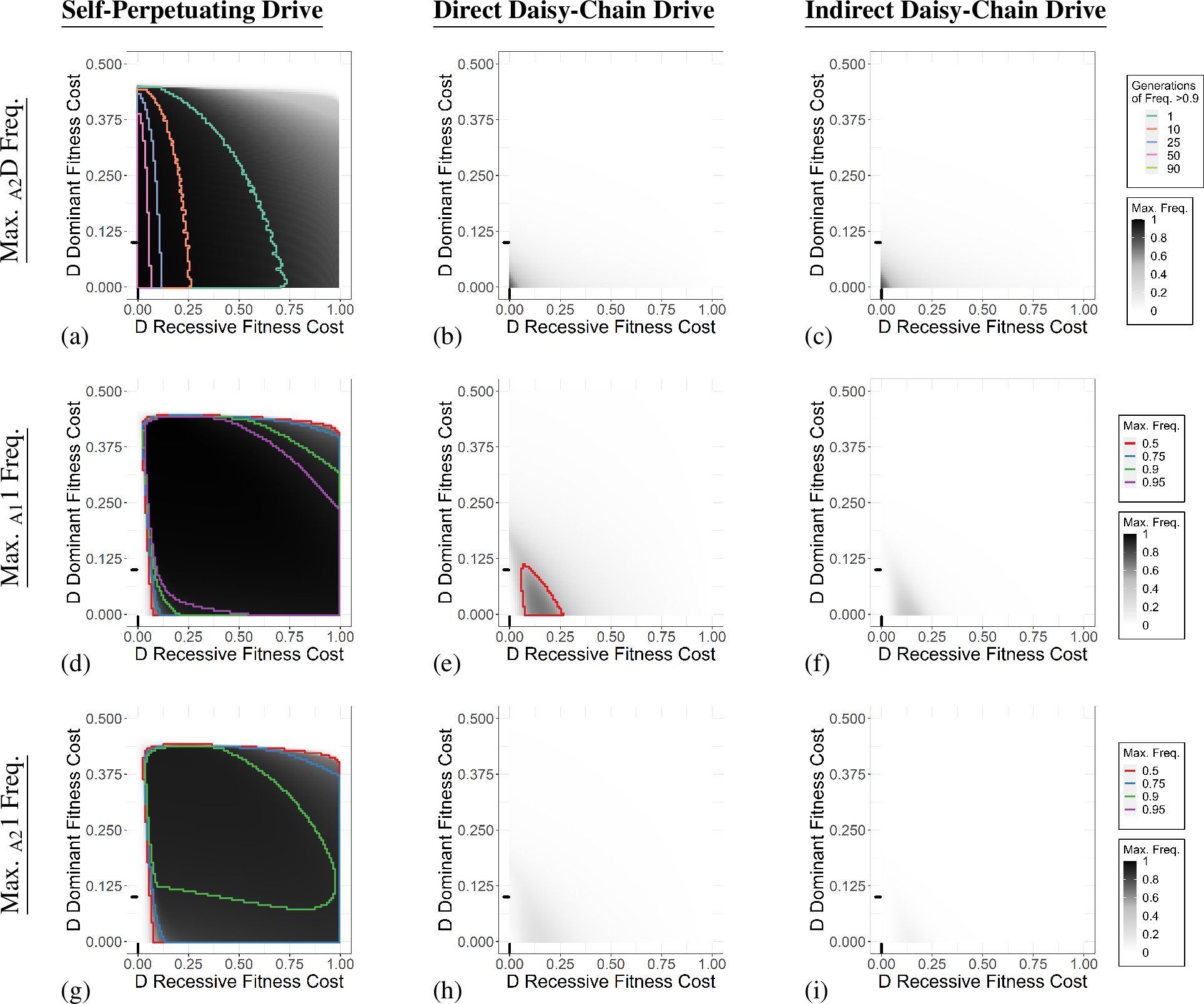

Supplement: S7 Fig — Row 1 (a-c). Maximum AD allele frequency in population two. Row 2 (d-f). Maximum A1 allele frequency in population one. Row 3 (g-i). Maximum A1 allele frequency in population two. The maximum frequency of AD in population one and the difference between population one and two is shown in Fig 2. (TIF) [file pgen.1010370.s007.tif]

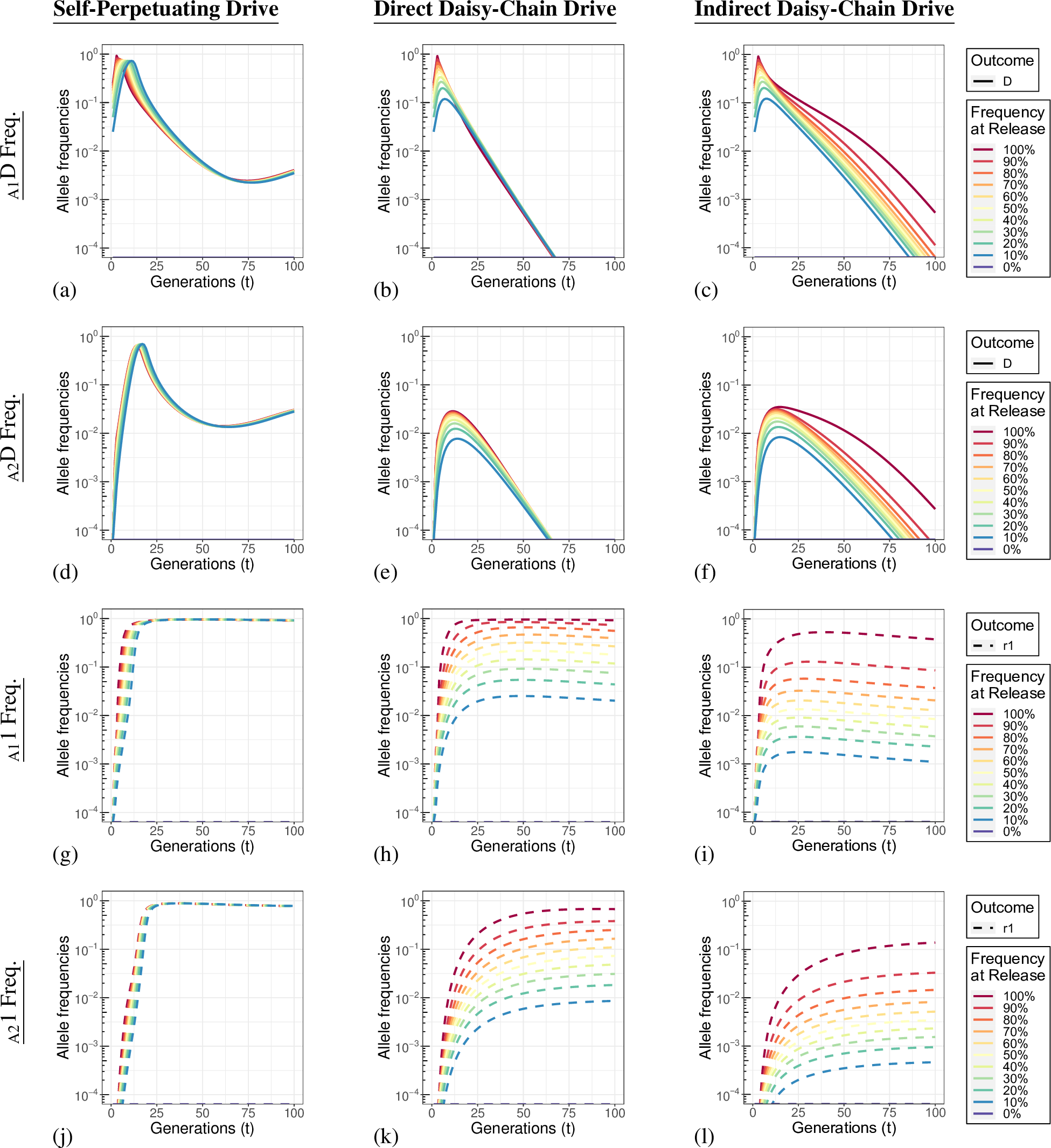

Supplement: S8 Fig — Row 1 (a-c). Allele dynamics of the A locus drive element in population one. Row 2 (d-f). Allele dynamics of the A locus drive element in population two. Row 3 (g-i). Allele dynamics of the A locus type-1 resistance mutations in population one. Row 4 (j-l). Allele dynamics of the A locus type-1 resistance mutations in population two. Column 1. Self-Perpetuating Drive. Column 2. Direct Daisy-Chain Drive. Column 3. Indirect Daisy-Chain Drive. A heterozygous release frequency of 100% means that all males in population one are heterozygous drive carriers at the start of the simulation. This equates to a drive allele frequency of 50% among males and a frequency of 25% among all alleles when females are included (the y-axis value shown). (TIF) [file pgen.1010370.s008.tif]

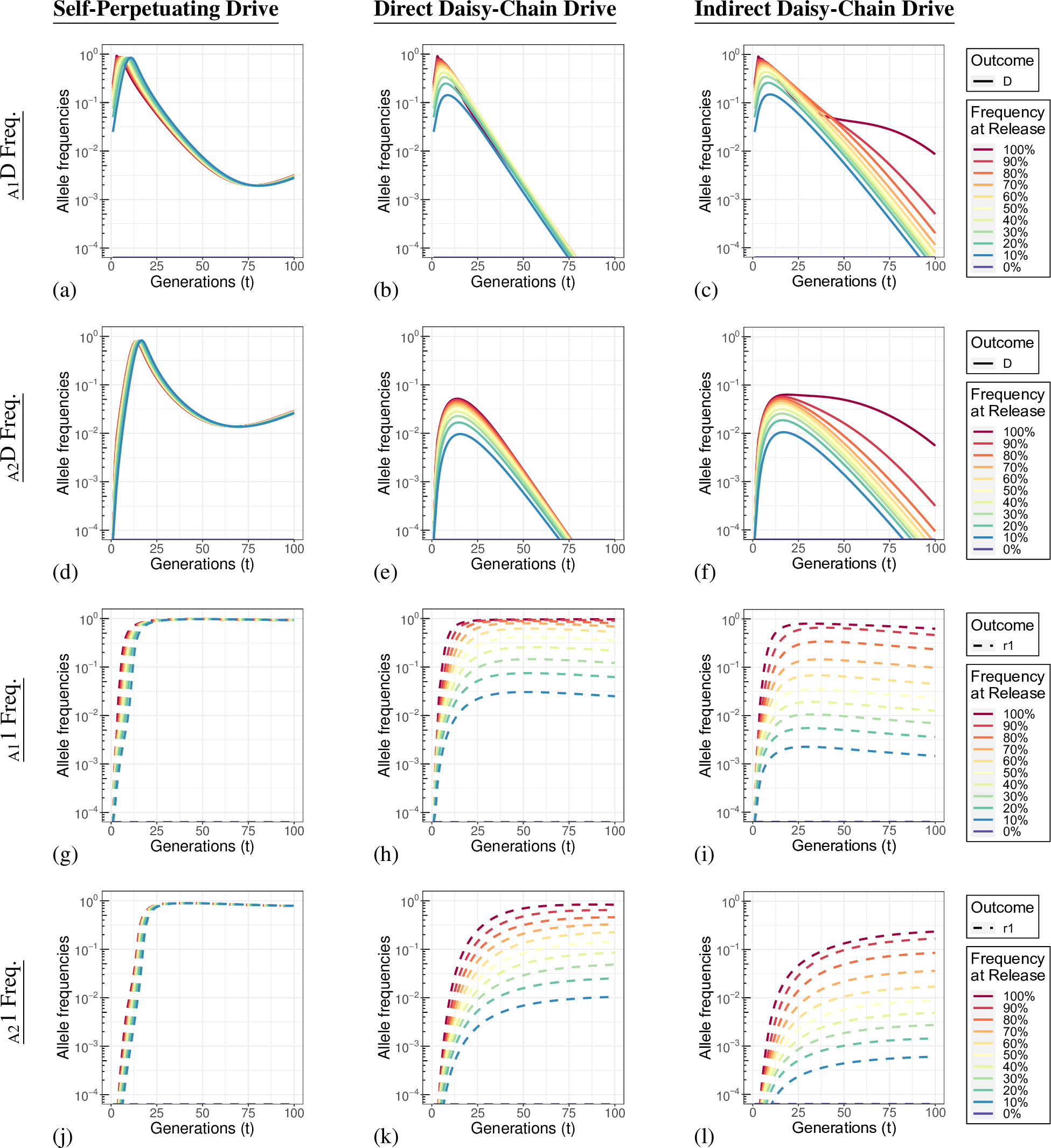

Supplement: S9 Fig — Row 1 (a-c). Allele dynamics of the A locus drive element in population one. Row 2 (d-f). Allele dynamics of the A locus drive element in population two. Row 3 (g-i). Allele dynamics of the A locus type-1 resistance mutations in population one. Row 4 (j-l). Allele dynamics of the A locus type-1 resistance mutations in population two. Column 1. Self-Perpetuating Drive. Column 2. Direct Daisy-Chain Drive. Column 3. Indirect Daisy-Chain Drive. A heterozygous release frequency of 100% means that all males in population one are heterozygous drive carriers at the start of the simulation. This equates to a drive allele frequency of 50% among males and a frequency of 25% among all alleles when females are included (the y-axis value shown). (TIF) [file pgen.1010370.s009.tif]

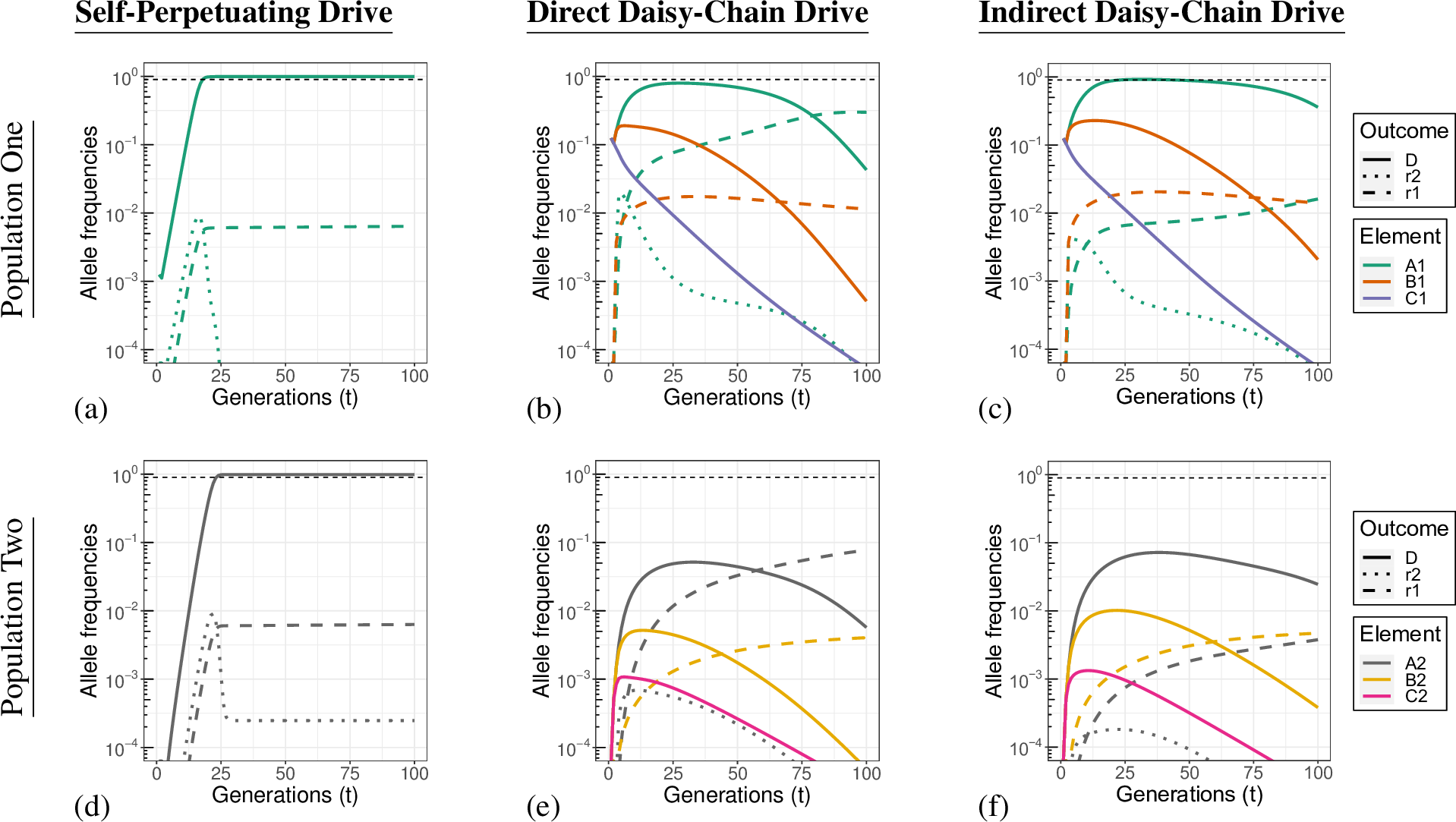

Supplement: S10 Fig — Row 1 (a-c). Individual allele dynamics for population one. Row 2 (d-f). Individual allele dynamics for population two. Column 1. Self-Perpetuating Drive. Column 2. Direct Daisy-Chain Drive. Column 3. Indirect Daisy-Chain Drive. The thin dashed line indicates a frequency of 90%. (TIF) [file pgen.1010370.s010.tif]

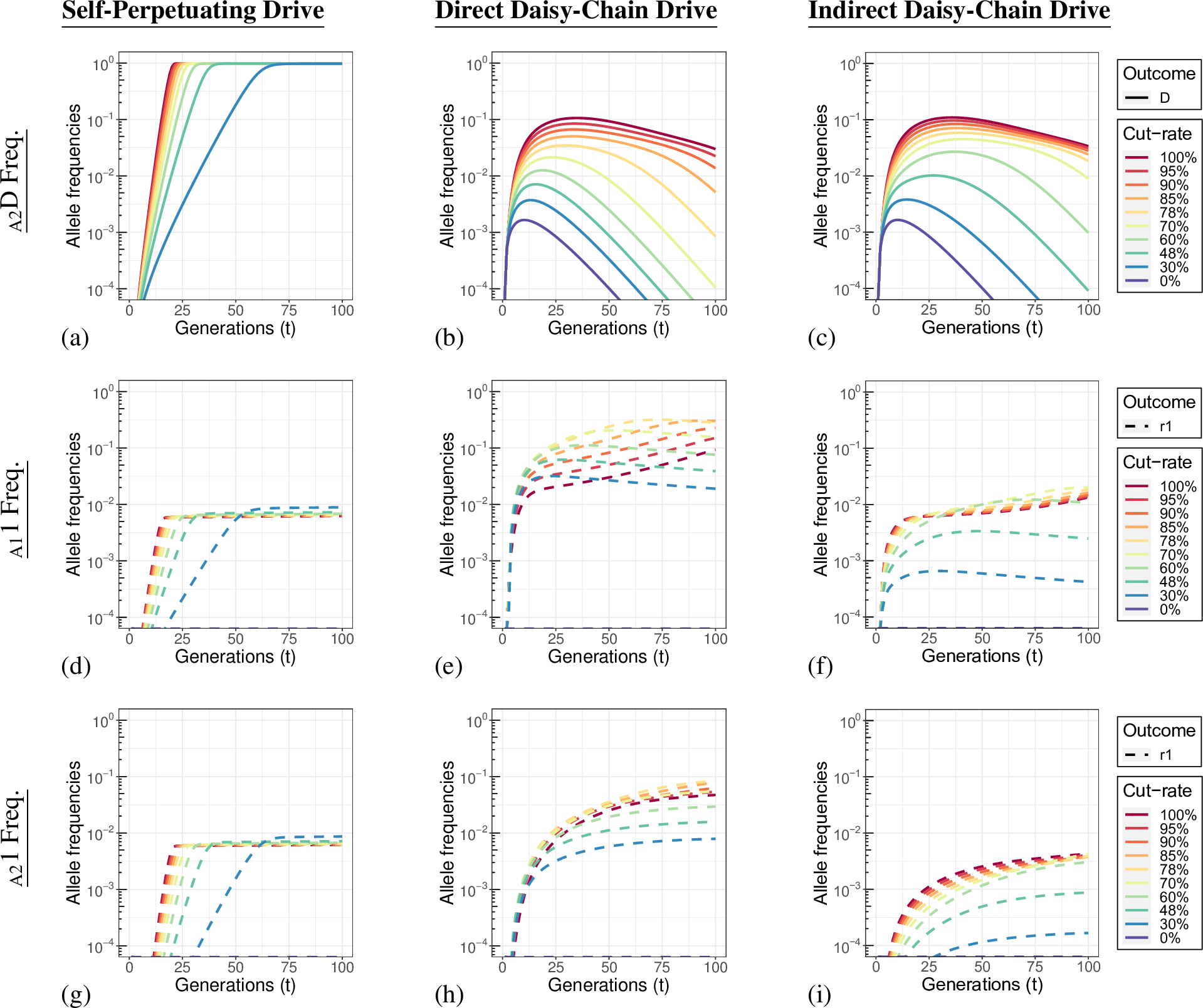

Supplement: S11 Fig — Row 1 (a-c). Allele dynamics of the drive element at the A locus in population two. Row 2 (d-f). Allele dynamics of type-1 resistance mutations at the A locus in population one. Row 3 (g-i). Allele dynamics of type-1 resistance mutations at the A locus in population two. Allele dynamics of the drive element at the A locus in population one are shown in Fig 3d–3f. Column 1. Self-Perpetuating Drive. Column 2. Direct Daisy-Chain Drive. Column 3. Indirect Daisy-Chain Drive. (TIF) [file pgen.1010370.s011.tif]

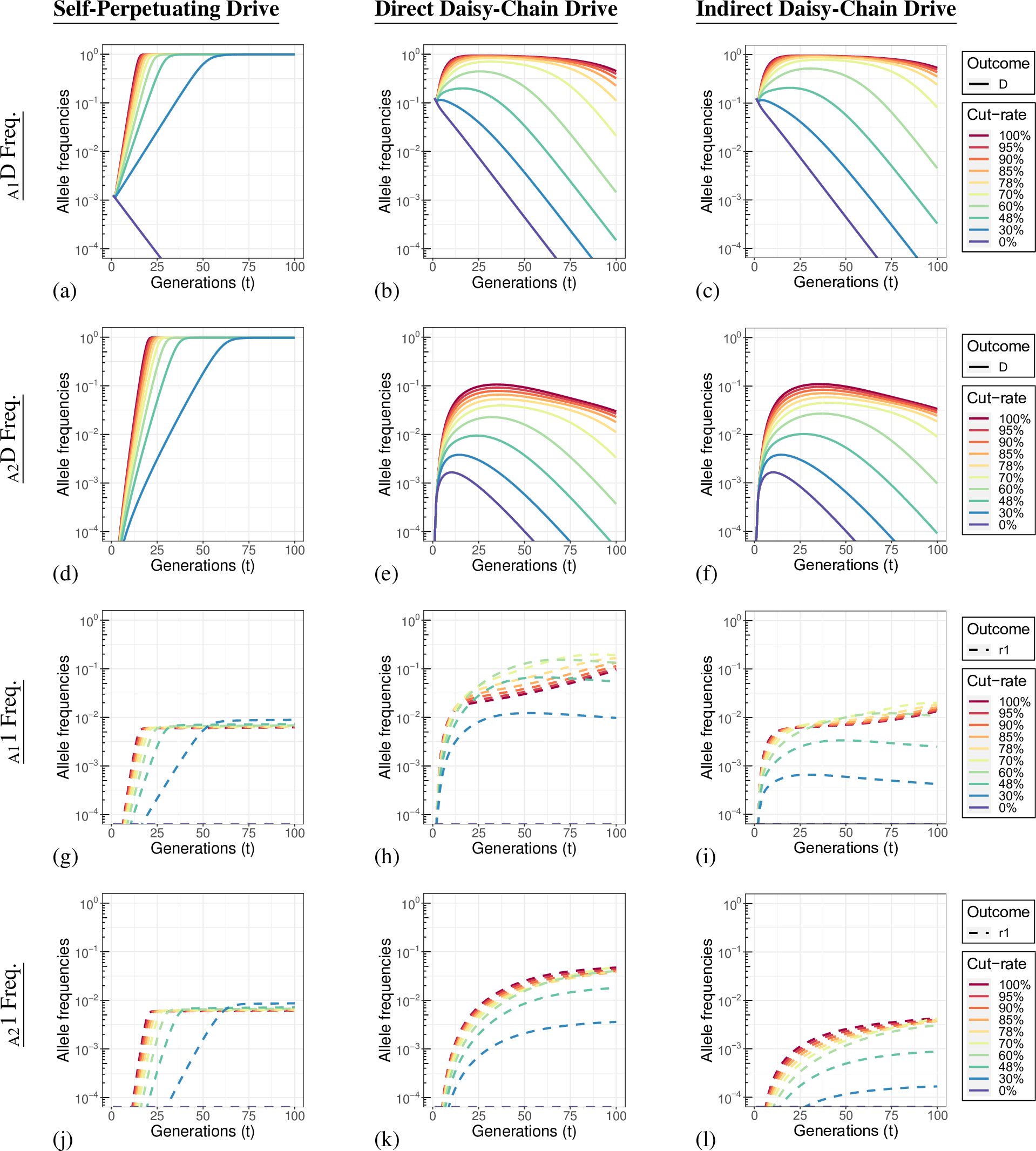

Supplement: S12 Fig — Row 1 (a-c). Allele dynamics of the A locus drive element in population one. Row 2 (d-f). Allele dynamics of the A locus drive element in population two. Row 3 (g-i). Allele dynamics of the A locus type-1 resistance mutations in population one. Row 4 (j-l). Allele dynamics of the A locus type-1 resistance mutations in population two. Column 1. Self-Perpetuating Drive. Column 2. Direct Daisy-Chain Drive. Column 3. Indirect Daisy-Chain Drive. (TIF) [file pgen.1010370.s012.tif]

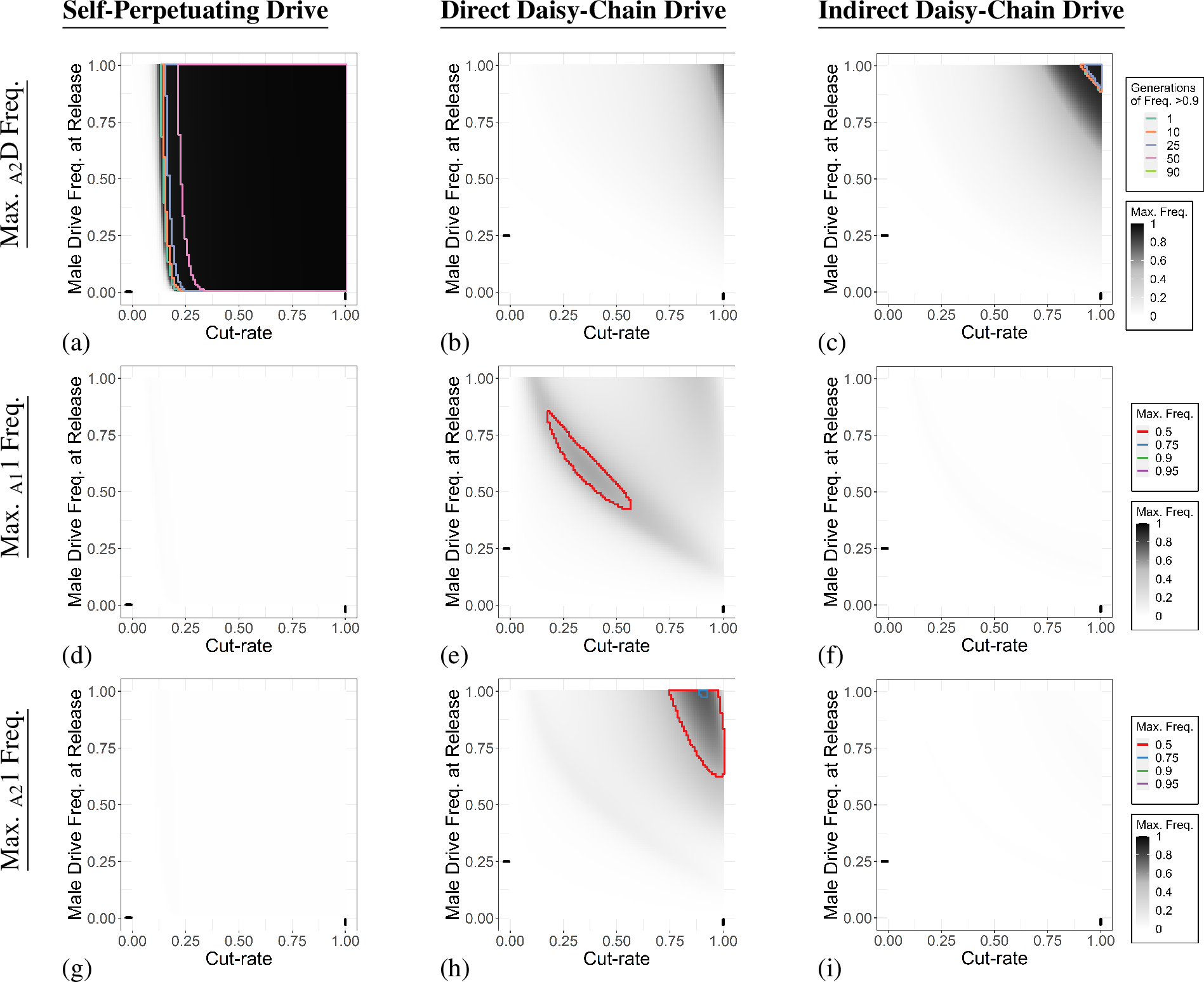

Supplement: S13 Fig — Row 1 (a-c). Maximum AD allele frequency in population two. Row 2 (d-f). Maximum A1 allele frequency in population one. Row 3 (g-i). Maximum A1 allele frequency in population two. The maximum frequency of AD in population one and the difference between population one and two is shown in Fig 3. (TIF) [file pgen.1010370.s013.tif]

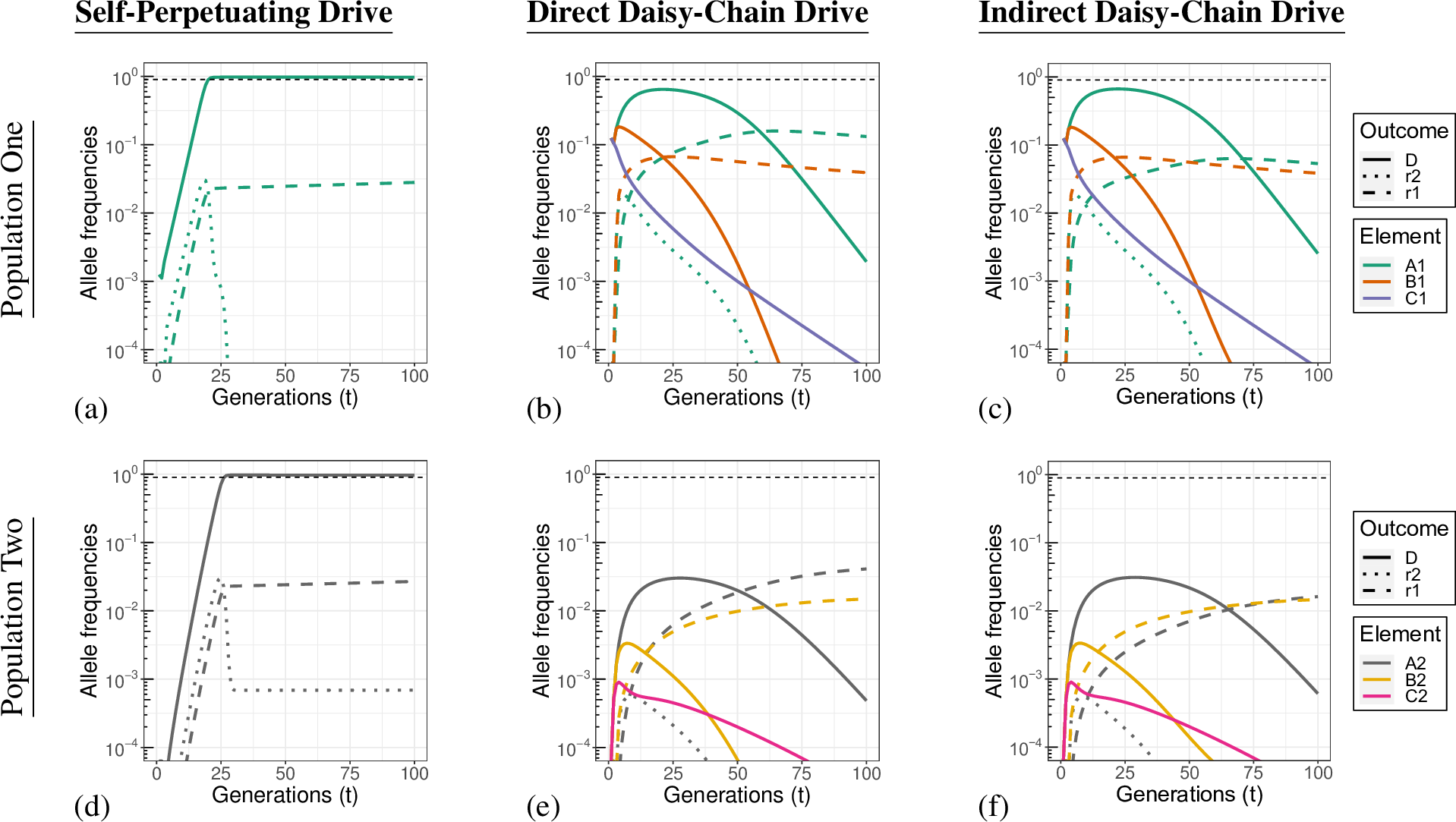

Supplement: S14 Fig — Row 1 (a-c). Individual allele dynamics for population one. Row 2 (d-f). Individual allele dynamics for population two. Column 1. Self-Perpetuating Drive. Column 2. Direct Daisy-Chain Drive. Column 3. Indirect Daisy-Chain Drive. HDR does not occur with deposition-mediated cutting, and all cuts result in resistance mutations following the 1:9 ratio of type-1 to type-2. The thin dashed line indicates a frequency of 90%. The dynamics of the direct and indirect DCD B1D elements may be more similar than initially expected from the genetics. However, when the DCD B elements are together with a drive element at A, there is no difference in deposition between the direct (AE/BNA) and indirect (AGA/BN) DCDs. Only when the B element is isolated from the A drive element does deposition affect the two designs differently. This only occurs when the B element segregates away with a type-1 resistance allele (100% expression based cut-rate, and type-2 resistance alleles are lethal). Although the resistance allele rate is substantially higher with deposition, isolation of BD from AD is still a rare event. Moreover, due to the high AD frequency, isolated B elements are rapidly reacquainted with a A drive element. Isolated BD elements are much less likely to be reacquainted with a AD element in population two, which is reflected in a more pronounced difference in BD allele dynamics between the indirect and direct DCDs. (TIF) [file pgen.1010370.s014.tif]

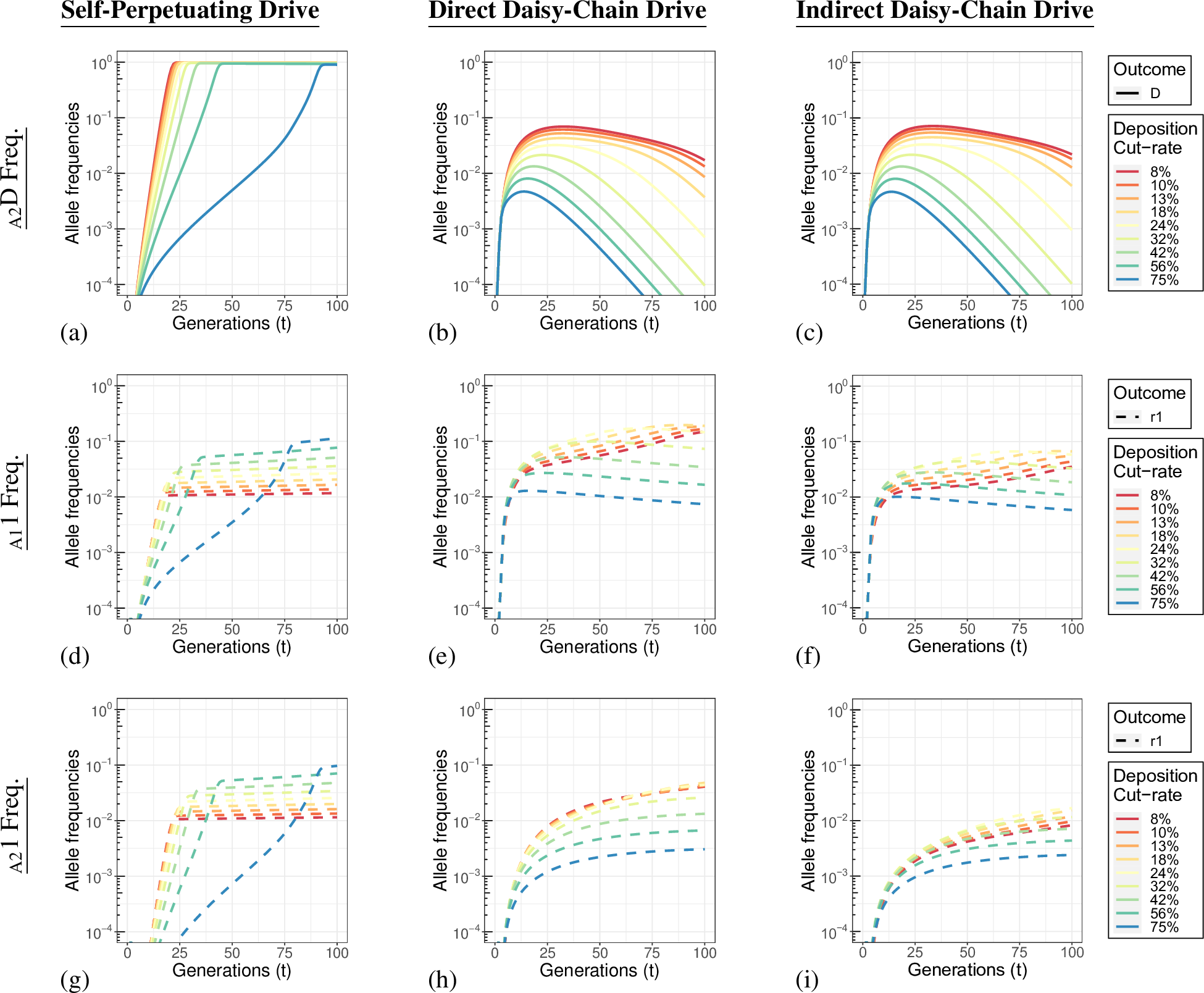

Supplement: S15 Fig — Row 1 (a-c). Allele dynamics of the drive element at the A locus in population two. Row 2 (d-f). Allele dynamics of type-1 resistance mutations at the A locus in population one. Row 3 (g-i). Allele dynamics of type-1 resistance mutations at the A locus in population two. Allele dynamics of the drive element at the A locus in population one are shown in Fig 4d–4f. Column 1. Self-Perpetuating Drive. Column 2. Direct Daisy-Chain Drive. Column 3. Indirect Daisy-Chain Drive. HDR does not occur with deposition-mediated cutting, and all cuts result in resistance mutations following the 1:9 ratio of type-1 to type-2. (TIF) [file pgen.1010370.s015.tif]

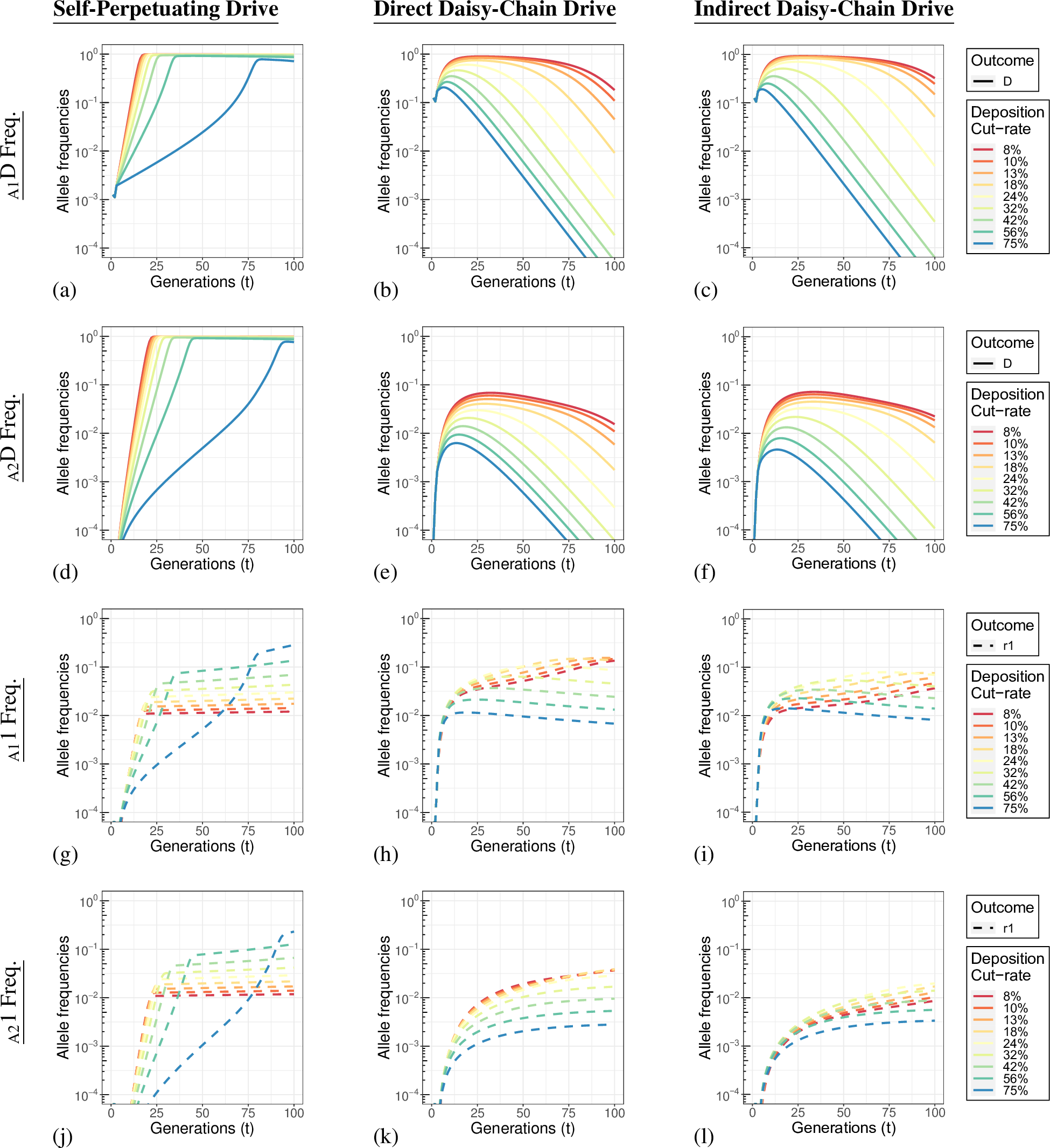

Supplement: S16 Fig — In these simulations only the Cas9 protein is deposited. Row 1 (a-c). Allele dynamics of the A locus drive element in population one. Row 2 (d-f). Allele dynamics of the A locus drive element in population two. Row 3 (g-i). Allele dynamics of the A locus type-1 resistance mutations in population one. Row 4 (j-l). Allele dynamics of the A locus type-1 resistance mutations in population two. Column 1. Self-Perpetuating Drive. Column 2. Direct Daisy-Chain Drive. Column 3. Indirect Daisy-Chain Drive. HDR does not occur with deposition-mediated cutting, and all cuts result in resistance mutations following the 1:9 ratio of type-1 to type-2. (TIF) [file pgen.1010370.s016.tif]

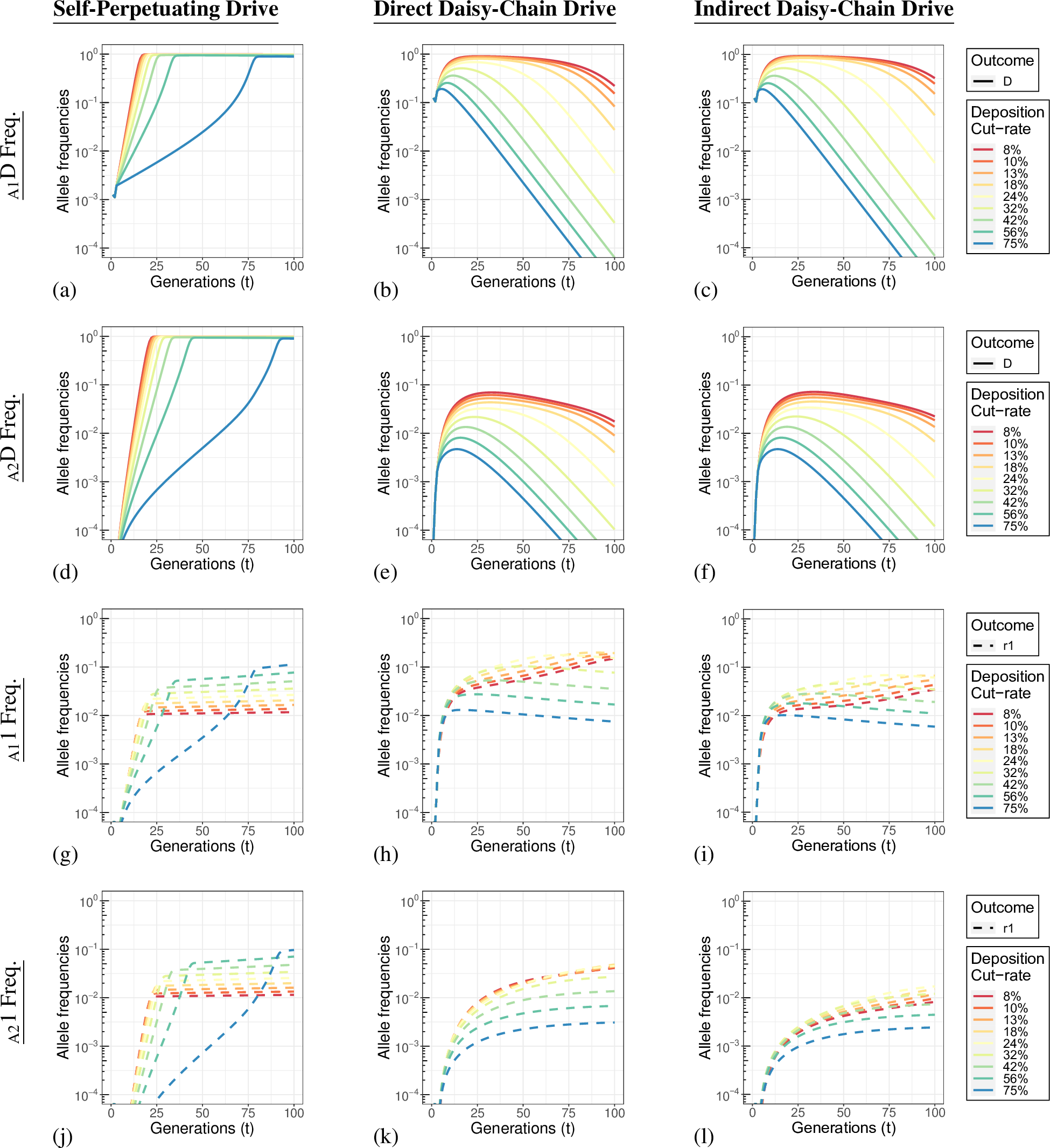

Supplement: S17 Fig — In these simulations, when the deposited Cas9 protein is paired with an expressed gRNA, DNA repair uses the germline HDR rate. If Cas9 and a gRNA are deposited simultaneously, this takes precedence, and HDR repair is not possible. In practise, shadow drive occurs only when the mother carries the Cas9 and the father provides the gRNA gene. Note that this scenario cannot occur with the self-perpetuating drive as the Cas9 and gRNA gene are always linked. Row 1 (a-c). Allele dynamics of the A locus drive element in population one. Row 2 (d-f). Allele dynamics of the A locus drive element in population two. Row 3 (g-i). Allele dynamics of the A locus type-1 resistance mutations in population one. Row 4 (j-l). Allele dynamics of the A locus type-1 resistance mutations in population two. Column 1. Self-Perpetuating Drive. Column 2. Direct Daisy-Chain Drive. Column 3. Indirect Daisy-Chain Drive. (TIF) [file pgen.1010370.s017.tif]

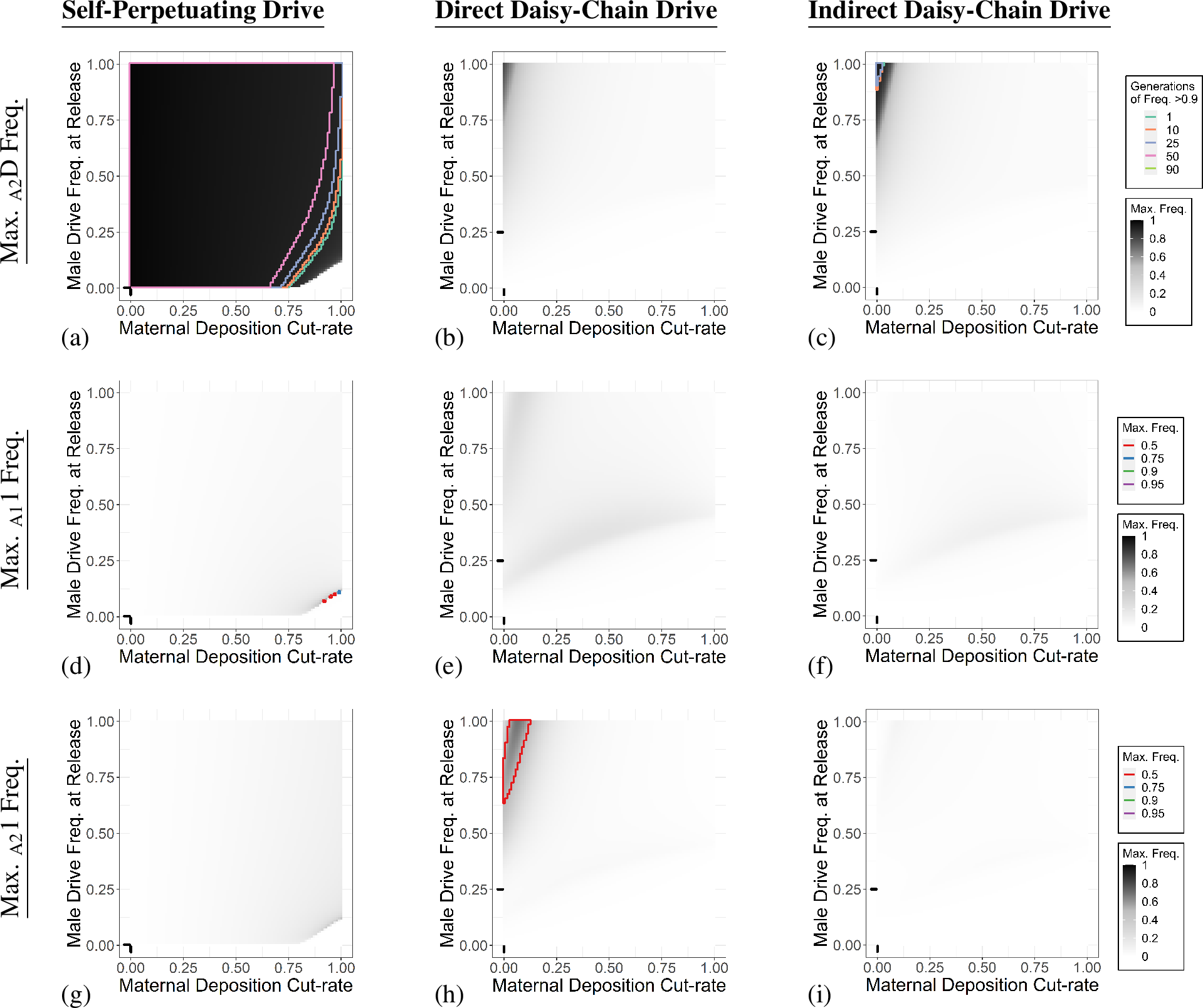

Supplement: S18 Fig — Row 1 (a-c). Maximum AD allele frequency in population two. Row 2 (d-f). Maximum A1 allele frequency in population one. Row 3 (g-i). Maximum A1 allele frequency in population two. The maximum frequency of AD in population one and the difference between population one and two is shown in Fig 4. HDR does not occur with deposition-mediated cutting, and all cuts result in resistance mutations following the 1:9 ratio of type-1 to type-2. (TIF) [file pgen.1010370.s018.tif]
